# Coupled Molecular and Framework Descriptors for Band-Edge Spin Splitting in Chiral Hybrid Organic–Inorganic Perovskites

**DOI:** 10.1021/jacs.6c11593

**Published:** 2026-08-26

**Authors:** Muskan Nabi, Juan J. Aucar, Xinfeng Chen, Gustavo A. Aucar, Alessandro Stroppa

**Affiliations:** † CNR-SPIN, Department of Physical and Chemical Sciences, University of L’Aquila, c/o - Via Vetoio, Coppito, L'Aquila 67100, Italy; ‡ Institute for Modelling and Innovative Technology, IMIT (CONICET-UNNE), Avda Libertad 5460, W3404AAS Corrientes, Argentina; § Key Lab of Advanced Optoelectronic Quantum Architecture and Measurement (MOE), Beijing Key Laboratory of Quantum Matter State Control and Ultra-Precision Measurement Technology, and School of Physics, Beijing Institute of Technology, Beijing 100081, China

## Abstract

Chiral hybrid organic–inorganic perovskites offer a chemically tunable platform in which inversion-asymmetric crystal fields and strong spin–orbit coupling generate spin-split band edges, yet the mechanisms through which the chiral molecules and the inorganic framework interact to produce spin splitting are not well understood. Here we use the electronic chirality measure—a wave function-based metric—to quantify molecular chirality across three substitution series of chiral metal halides derived from methylbenzylammonium ligands. We find that halogen functionalization increases the valence-band spin splitting from 6.6 meV in the parent system to 64.8 meV in the benzylic-halogenated (S-MBA)­X series and up to 110.0 meV in the side-chain-halogenated (S-MBA)­CH_2_X series (X = H, F, Cl, Br). In contrast, for the pnictogen-substituted (S-MBA)Y series (Y = P, As, Sb, Bi), the splitting remains in the few-meV regime. The electronic chirality measure captures changes in the chiral electronic structure and provides a useful molecular descriptor for comparison with parity-violating energy differences, but it does not alone determine the band-edge splitting. The largest splittings emerge when electronic chirality, intrinsic cation dipoles, and inorganic-framework distortion act cooperatively. Within the present substitution series, larger intrinsic cation dipoles are associated with smaller spin splitting energies. Large band-edge splitting occurs only when molecular chirality is efficiently converted into the induced chiral Pb/I framework mode. We identify the chirality transfer efficiency (CTE), a descriptor introduced in this work, together with the cation electrostatic environment, as key parameters governing chirality transfer.

## Graphical abstract

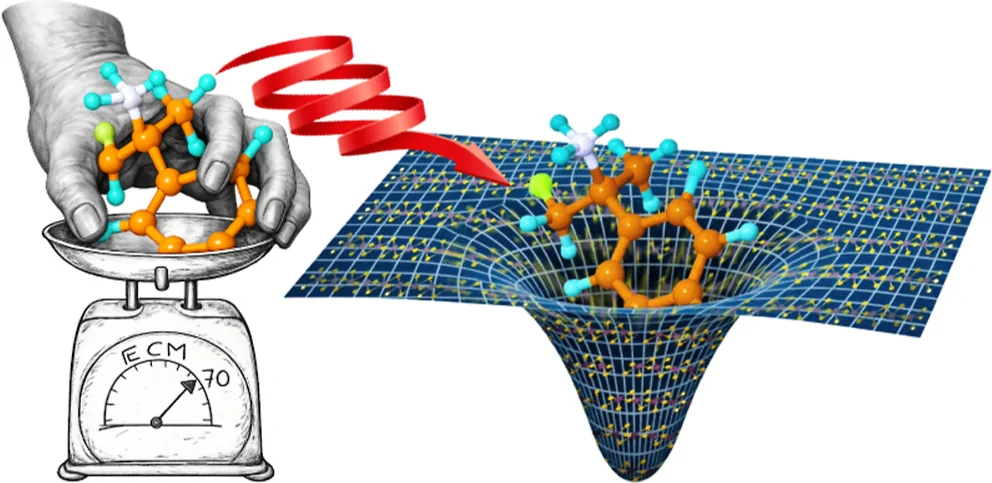

## Introduction

Chirality is a unifying concept across chemistry, materials science, and condensed matter physics, where it governs optical activity, circular dichroism (CD), nonlinear optical responses, ferroelectricity, and spin-dependent transport properties.^1–4^ An object is chiral if it cannot be superimposed on its mirror image by any combination of proper rotations and translations, which is equivalent to lacking any improper symmetry operation (reflection, inversion, rotoinversion, or glide reflection).^5^

Hybrid organic–inorganic perovskites (HOIPs) provide a platform in which chiral organic cations act as molecular “knobs” that imprint chirality on the crystal. Through chirality transfer (CT),^6–8^ molecular asymmetry can induce symmetry-breaking distortions of the inorganic framework. Their structural and compositional flexibility allows structural and electronic properties to be tailored, with chiral signatures tunable through ligand choice, dimensionality control, and external stimuli.^9^ Since the band-edge electronic states are largely derived from the inorganic framework, these cations can indirectly influence the band structure and the associated spin-optoelectronic response. Quantifying the intrinsic chiral strength of the molecular cations and the efficiency of its propagation to the inorganic framework is therefore important for developing design rules to improve spin-optoelectronic performance.

A consistent measure of chirality in complex materials requires descriptors that go beyond geometric structure alone.^10,11^ The electronic chirality measure (ECM)^12^ addresses this need by defining an inner product over electronic wave functions, comparing a chiral molecule’s electron distribution to that obtained by a rigid shift of such wave function to the nearest achiral configuration. This construction provides a quantitative measure of molecular chirality that reflects both nuclear geometry and electronic structure. Recent work reported an interdependence between ECM and parity-violating (PV) energy differences (Δ*E* ^PV^),^13^ linking ECM to fundamental weak interactions. Implemented within a four-component relativistic formalism,^14^ ECM treats electronic states and ionic degrees of freedom on an equal footing, complementing geometry-based metrics that have been proposed in the literature.^11,15–20^ In principle, this molecular descriptor can support the rational analysis of chirality-dependent phenomena, such as chirality-induced spin selectivity (CISS),^21–23^ by connecting the molecular source of chirality to the solid-state response.^24,25^

In HOIPs, broken inversion symmetry combined with spin–orbit coupling (SOC) can generate sizable spin splitting, which is relevant for spintronic applications.^9,26–30^ Although chirality in HOIPs is routinely assessed by CD, VCD, optical rotation,^31,32^ these observables probe macroscopic chiroptical response and do not directly isolate the local electronic chirality and framework-level contributions that drive SOC-induced band-edge splitting. Therefore, a quantitative picture connecting molecular chirality, its transfer to the inorganic framework, and the spin splitting of band states remains incomplete.

In this work we examine three chemically engineered substitution series of chiral hybrid perovskites. Using the electronic chirality measure, intrinsic cation dipoles, and spin–orbit-coupled first-principles calculations, we show that neither ECM nor dipole magnitude alone explains the observed trends. Instead, large Rashba-type splitting emerges from an appropriate interplay between molecular descriptors and framework distortion: halogen functionalization increases the Rashba-type band-edge spin splitting energy scale *E*_R_ from 6.6 meV in the parent material to as much as 110.0 meV, whereas pnictogen substitution leaves *E*_R_ in the few-meV regime. The series are therefore not intended to define a single substitution coordinate. Rather, they provide three internally consistent computational perturbation classes: benzylic halogenation, cavity-facing side-chain halogenation, and pnictogen substitution. These correspond to the benzylic-halogenated (S-MBA)­X series, the side-chain-halogenated (S-MBA)­CH_2_X series, and the pnictogen-substituted (S-MBA)Y series (X = H, F, Cl, Br; Y = P, As, Sb, Bi). Framework-only symmetry-mode analysis further identifies the induced Γ_1_^–^ mode as the clearest structural fingerprint of chirality transfer in these materials, which quantifies the CT efficiency (CTE). The present work is a first-principles study of ideal ordered crystals; the predicted splittings and correlations should be viewed as computational design trends to be tested by spin-resolved spectroscopy or transport/chiroptical measurements.

### Descriptor Definitions and Computational Details

Spin–orbit-coupled density functional theory (DFT) calculations were performed to extract band-edge spin splitting in the relaxed crystals, while the ECM, Δ*E* ^PV^, and intrinsic cation dipoles were evaluated for the corresponding isolated cations extracted from the relaxed structures. Full definitions of the descriptors and the dipole convention for charged molecules are provided in the Supporting Information. In the relativistic regime, parity-violating contributions to molecular energies are dominated by the nuclear spin-independent effective Hamiltonian.^33–35^ For achiral molecules the PV contribution to the total energy vanishes, whereas for a pair of enantiomers it changes sign, giving rise to the energy difference Δ*E* ^PV^,^36^ which is governed by the weak interaction that occurs within the nuclei, between electrons and nucleons, and is strongly enhanced for heavy atoms. The properties of SOC-split bands, associated with Pb/I-derived band-edge states in a noncentrosymmetric crystal field, are closely linked to electric polarization,^37,38^ including organohalide perovskites where spin splitting may be influenced by intrinsic unit-cell dipoles.^39^ Motivated by recent work on dipole tuning through *para*-halogenation of neutral ligands,^40^ we focus here on the magnitude of the intrinsic electric dipole moment (|**p**|) of the organic cations and systematically explore multiple chemical modifications. To compare dipole moments consistently for charged cations,^41^ we adopt a local reference frame^42^ centered at the barycenter of the positive charges. This cation-centered convention fixes a common local origin and yields a consistent intrinsic dipole descriptor that reflects the separation between the barycenters of positive and negative charge (see Supporting Information, Section “Electric Dipole Moment”). Throughout this work, |**p**| denotes the magnitude of the intrinsic electric dipole moment of the isolated organic cation extracted from the relaxed crystal geometry, and is reported in atomic units (a.u.). The SOC term can be written in terms of the local inversion-asymmetric electric field (see eqs S7 and S18), while the leading dipolar contribution from the organic cation is summarized in eq S10.

Our first-principles calculations were performed using DFT as implemented in the Vienna Ab initio Simulation Package (VASP).^43–45^ We adopted the Perdew–Burke–Ernzerhof (PBE) exchange–correlation functional within the generalized gradient approximation (GGA).^46^ A plane-wave basis set within the projector augmented wave (PAW) method^47,48^ was employed, using an energy cutoff of 550 eV. van der Waals (vdW) interactions were described using the DFT-D3 method developed by Grimme.^49^ The Brillouin zones were sampled with the Monkhorst–Pack scheme, using 8 × 8 × 4 *k*-point grids. The energy convergence criterion was set to 10^–5^ eV and the Hellmann–Feynman forces criterion during structural relaxation was 0.01 eV/Å. The SOC effect was included self-consistently during the electronic structure calculations.^50^ Details of the symmetry-mode analysis, performed with the Bilbao Structure Relations/PSEUDO tools and AMPLIMODES,^51–53^ are provided in the Supporting Information, Section “Structural Analysis of the Relaxed Crystals”.

The ECM descriptor, Δ*E* ^PV^, and the electric dipole moments of the organic cations were computed at the X2C-PBE0 level, including spin-free and spin-dependent contributions. ECM values were obtained with the PyECM26 ^54^ code on top of PySCF wave functions,^55,56^ using nearest achiral configurations generated with CoSyM.^57^ PV energy differences and dipole moments were calculated with DIRAC^58,59^ and Dyall’s uncontracted relativistic triple-ζ (cv3z) basis sets.^60^ Molecular geometries were extracted directly from the optimized solid-state structures without further reoptimization.

## Results and Discussion

We examine how targeted organic-cation substitutions modify molecular properties and lattice asymmetry, and how these changes influence spin splitting in chiral HOIPs.

### Structural Details

To tune the organic-cation chemistry while preserving the parent topology, we introduce targeted substitutions across three HOIP series. This strategy, previously shown to enhance chiroptical properties and spin splitting in related systems,^61,62^ allows us to probe how controlled electronic and steric modifications of the cation affect CT and, ultimately, spin splitting within a common structural backbone. This design belongs to a broader, rapidly expanding paradigm of chiral HOIP engineering. Our calculated trends closely parallel emerging literature benchmarks. For example, targeted peripheral ligand modifications—such as aromatic halogenation and systematic substitution profiles—can substantially amplify chiroptical anisotropy profiles without disrupting the core host framework topology.^63–65^ Moreover, the underlying framework symmetry and the strength of the ligand-to-framework coupling act as the primary mediators of this solid-state response, heavily governed by the intricate noncovalent bonding and interfacial networks that transfer structural distortions into the inorganic cage.^66–69^

Specifically, we investigate three substitution series, represented by the prototype compounds (S-MBA)­PbI_3_, (S-MBA)­CH_3_PbI_3_, and (S-MBA)­PPbI_3_, respectively, as schematically illustrated in Figure 1. Here, MBA denotes the methylbenzylammonium cation, and the prefix “S-” specifies the absolute configuration of the chiral center.^70^ In the (S-MBA)­X series, benzylic halogenation is introduced by replacing a selected H atom with X = F, Cl, or Br, allowing us to probe how local halogen functionalization modifies chirality transfer and framework distortion within a common ligand scaffold. All structures were generated starting from the known prototype (S-MBA)­PbI_3_.^62^ In the (S-MBA)­CH_2_X series, a hydrogen atom in the side-chain methyl group of (S-MBA)­CH_3_ is replaced by a halogen, providing a distinct substitution geometry that couples more directly to the inorganic cavity. The pnictogen-substituted (S-MBA)­Y series serves as a descriptor-based computational control, rather than as a claim of immediate synthetic accessibility for all pnictogen analogues. This design allows us to assess the influence of the central atom’s electronic properties and polarizability on CT, thereby separating intrinsic electronic effects from the steric contributions that dominate in the halogenated series.

**Figure 1:**
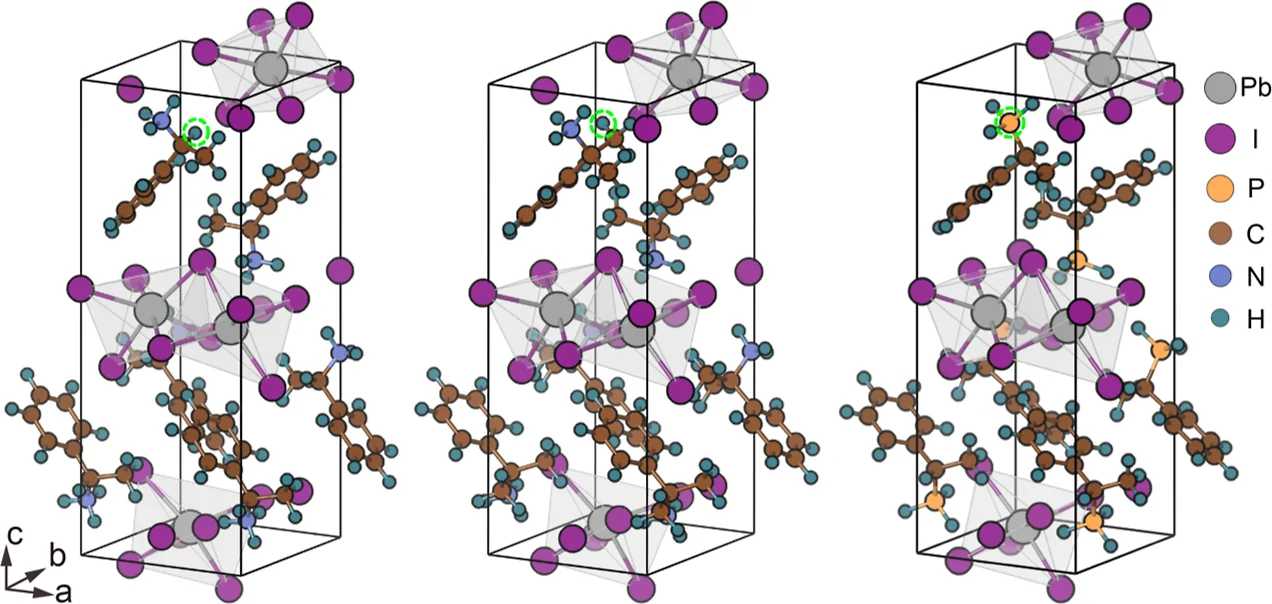
Crystal structures of (A) (S-MBA)­PbI_3_, (B) (S-MBA)­CH_3_PbI_3_, and (C) (S-MBA)­PPbI_3_, used as representative prototypes for the three substitution series considered in this work. The replaced atoms are highlighted with green dashed circles in one of the organic molecules.

All optimized structures retain the orthorhombic chiral Sohncke space group *P*2_1_2_1_2_1_ (No. 19) after full relaxation; the lattice parameters are reported in Table S1 in the Supporting Information. Here, chiral refers both to the crystallographic space group, which contains only proper symmetry operations, including three mutually perpendicular 2_1_ screw rotations, and lacks any improper symmetry elements such as inversion centers, mirror planes, glide planes, or rotoinversion axes; and to the definite handedness imposed by the enantiopure (S)-MBA-derived cations on the crystal structure that transfers chirality to the inorganic framework through such induced structural distortion. As shown in Figure 1, the structures contain a perovskite-derived one-dimensional Pb/I framework built from face-sharing PbI_6_ octahedra, separated by chiral organic cations. Variations in the organic cation modulate the distortions of the octahedral units and therefore influence the electronic structure.^66^ For compactness, compound labels in tables refer to the organic cation; all crystals contain the corresponding Pb/I inorganic framework.

The three series affect the PbI_6_ framework in qualitatively different ways. The (S-MBA)­X series produces an intermediate structural response: fluorination yields the largest overall framework distortion, whereas the heavier halogens more strongly enhance bond-length inequivalence and Pb–I–Pb linkage-angle anisotropy. The (S-MBA)­CH_2_X series gives the strongest structural response. Relative to the CH_3_ reference, both octahedral distortion metrics and framework displacements increase sharply from CH_2_F to CH_2_Br, consistent with more efficient coupling between a cavity-facing substituent and the inorganic network. By contrast, the (S-MBA)Y series primarily modifies the lattice metrics, especially along *c*, while preserving a substantially more regular PbI_6_ framework.

To isolate the framework component of chirality transfer, we removed the organic molecules and decomposed the relaxed frameworks with respect to symmetrized parent structures using the Bilbao Structure Relations/PSEUDO tools and AMPLIMODES.^51–53^ In all three series the same symmetry-lowering pattern is obtained: two dominant achiral zone-boundary modes, *X*_4_^+^ and *X*_4_^–^, a negligible Γ_1_^+^ contribution, and a finite chiral zone-center mode Γ_1_^–^. This decomposition is consistent with a trilinear invariant

1 $$F_{tri}=\lambda Q_{X_{4}^{+}}Q_{X_{4}^{-}}Q_{\Gamma_{1}^{-}}$$

so that the induced Γ_1_^–^ mode serves as a symmetry-based structural measure of chirality transfer. The modes *X*_4_^+^, *X*_4_^–^, and Γ_1_^–^ are static, symmetry-adapted distortion modes obtained from a group-theoretical decomposition of the framework sublattice only, not phonon eigenmodes. Their amplitudes are extracted by projecting the relaxed framework geometry onto symmetry-adapted basis vectors using AMPLIMODES.^53^ In the halogenated series, the induced Γ_1_^–^ mode is dominated by opposite displacements of symmetry-related equatorial iodides, corresponding to a handed shear of the iodide sublattice, with only a small Pb counter-displacement. The *X*_4_^+^ and *X*_4_^–^ components are the dominant achiral distortions that provide the primary symmetry-breaking channel. The trilinear term in eq 1 is introduced as a symmetry-allowed Landau invariant to rationalize why the secondary chiral mode can be induced by coupling of two primary achiral distortions. It is not a fitted free-energy model; a meaningful energetic scan would require a full all-atom treatment beyond the scope of this work.

For the atomic displacement patterns associated with the different symmetry modes, we refer to the Supporting Information, where the participating atoms and the physical character of *X*_4_^+^, *X*_4_^–^, and Γ_1_^–^ are described explicitly. Table S3 reports the dominant coefficients of the normalized polarization vector for the induced Γ_1_^–^ mode, specifying the relative iodide (I) and lead (Pb) contributions for each compound. Figure S2 provides a visual comparison of the mode amplitudes $Q_{\Gamma_{1}^{-}}$ , $Q_{X_{4}^{+}}$ , $Q_{X_{4}^{-}}$ and *Q*_glob_ across all three substitution series.

Although the induced Γ_1_^–^ mode provides a quantitative fingerprint of chirality transfer, its role should not be interpreted as implying a ferroic chiral order parameter.^71^ In the two halogenated series, (S-MBA)­X and (S-MBA)­CH_2_X, the corresponding eigenvector is dominated by opposite displacements of symmetry-related equatorial iodides, accompanied by only a small Pb counter-displacement, showing that chirality transfer is encoded as a handed shear of the iodide sublattice rather than as a simple polar Pb off-centering.

The three substitution series differ primarily in how efficiently this induced chiral mode is amplified. In the (S-MBA)­X series, $Q_{\Gamma_{1}^{-}}$ increases monotonically from the parent framework to the F, Cl, and Br derivatives, even though the total distortion amplitude and the primary-mode sector are nonmonotonic. In the (S-MBA)­CH_2_X series, the amplification is markedly stronger and cooperative: $Q_{\Gamma_{1}^{-}}$ , $Q_{X_{4}^{+}}$ , and the global distortion amplitude all increase from CH_3_ to CH_2_Br. In the (S-MBA)­Y series, however, the induced chiral component remains strongly suppressed despite the persistence of a sizable *X*_4_^+^ sector. For the pnictogen-substituted series, the molecular substitution strongly changes the electronic structure of the cation, but couples much less efficiently to the cavity-facing iodide sublattice. As a result, the primary achiral framework response can remain finite while the conversion into the secondary chiral mode is weak. From a bonding/charge-distribution perspective, the perturbation is more strongly localized on the cation itself and less effectively projected onto the iodide displacements that build the chiral framework shear. The structural analysis therefore identifies three distinct regimes: selective amplification of the chiral mode in the first series, cooperative amplification in the second, and quenching of the induced chiral mode in the third.

From a structural standpoint, the key result is that local octahedral distortion alone does not uniquely rank chirality transfer. Large values of the bond length distortion (Δ*d*) or even of the global framework distortion can arise without equally efficient amplification of the induced chiral mode. What matters structurally is how effectively the primary achiral distortions feed the secondary Γ_1_^–^ mode that carries the chirality of the inorganic sublattice. This conversion is modest but systematic in the first series, strongest in the second, and largely suppressed in the pnictogen series. Local framework distortion is therefore a necessary condition, whereas the induced Γ_1_^–^ mode provides the more direct structural fingerprint of chirality transfer. Here, we introduce the chirality transfer efficiency, defined as $CTE=Q_{\Gamma_{1}^{-}}/Q_{glob}$ . It is not a universal material constant, but rather a normalized comparative descriptor within series that share the same parent framework and the same symmetry-mode decomposition. Its role is to measure which fraction of the total framework distortion is actually carried by the induced chiral mode. For this reason, CTE quantifies the efficiency with which the overall structural response is converted into the specific framework distortion that carries the inorganic handedness. Figure 2 shows the CTE for the systems under study, highlighting the selective amplification of the induced chiral mode in the (S-MBA)­X series, the cooperative amplification in the (S-MBA)­CH_2_X series, and the quenching in the (S-MBA)Y series (X = H, F, Cl, Br; Y = P, As, Sb, Bi). The final magnitude of the band-edge spin splitting must then reflect the combined action of this chiral framework component with the molecular electronic descriptors and the cation electrostatic environment discussed below.

**Figure 2:**
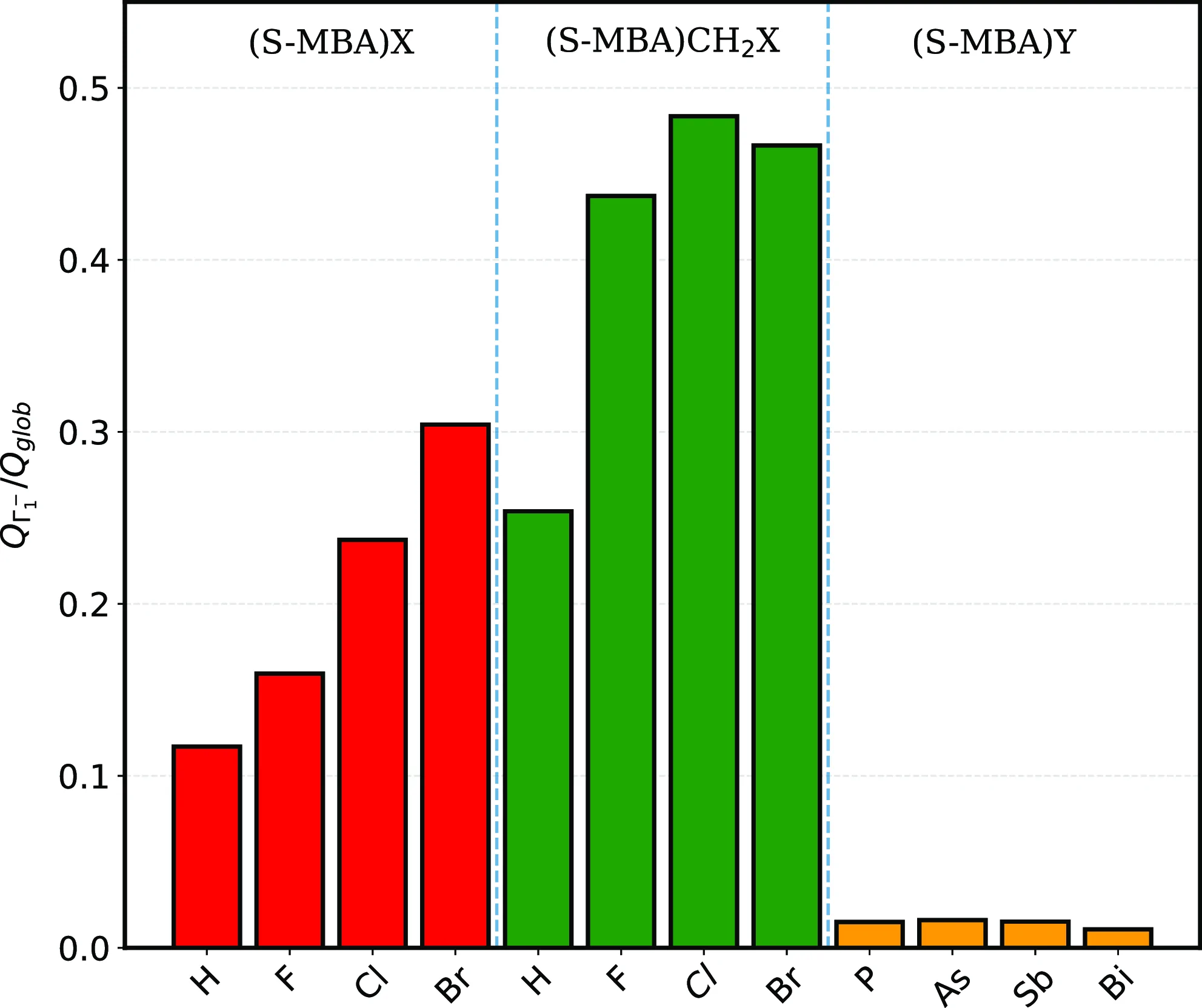
Structural efficiency of chirality transfer across the three substitution series. Bars report $Q_{\Gamma_{1}^{-}}/Q_{glob}$ , highlighting selective amplification of the induced chiral mode in the (S-MBA)­X series, cooperative amplification in the (S-MBA)­CH_2_X series, and quenching in the (S-MBA)­Y series (X = H, F, Cl, Br; Y = P, As, Sb, Bi).

The CTE is a framework-specific descriptor whose scope is limited to compounds that share the same parent framework. Its utility lies in identifying whether a given substitution amplifies or suppresses the chiral framework distortion relative to the total structural response, rather than in providing an absolute measure of chirality transfer that is transferable across unrelated framework topologies.

### SOC-Induced Band-Edge Spin Splitting

In the absence of inversion symmetry, SOC can lift the spin degeneracy of electronic bands, giving rise to momentum-dependent spin splitting.^72^ In the nonmagnetic systems considered here, time-reversal symmetry preserves Kramers degeneracy at time-reversal-invariant momenta (TRIM), while inversion breaking enables spin splitting away from those points. This manifests as band extrema shifted from high-symmetry points in the Brillouin zone and is relevant for spintronic phenomena such as spin Hall responses.

Figure 3 reports the band structures of the three series along the high-symmetry S–Y–S′ path. SOC is expected to be sizable due to the heavy elements (Pb and I). Along the S–Y–S′ path, the valence-band maximum (VBM) is displaced from Y, consistent with Rashba-type band-edge spin splitting. At the PBE + SOC level, the band gaps lie in the visible range (Table S4). In (S-MBA)­PbI_3_, SOC reduces the gap by about 0.4 eV relative to the calculation without SOC, consistent with previous work.^62^ The states near the band edges are predominantly Pb/I-derived in all three substitution series. Consequently, the effective SOC relevant for the spin splitting is governed mainly by the common Pb/I framework, while the molecular substitutions primarily modify the inversion-asymmetric crystal field experienced by these states.

**Figure 3:**
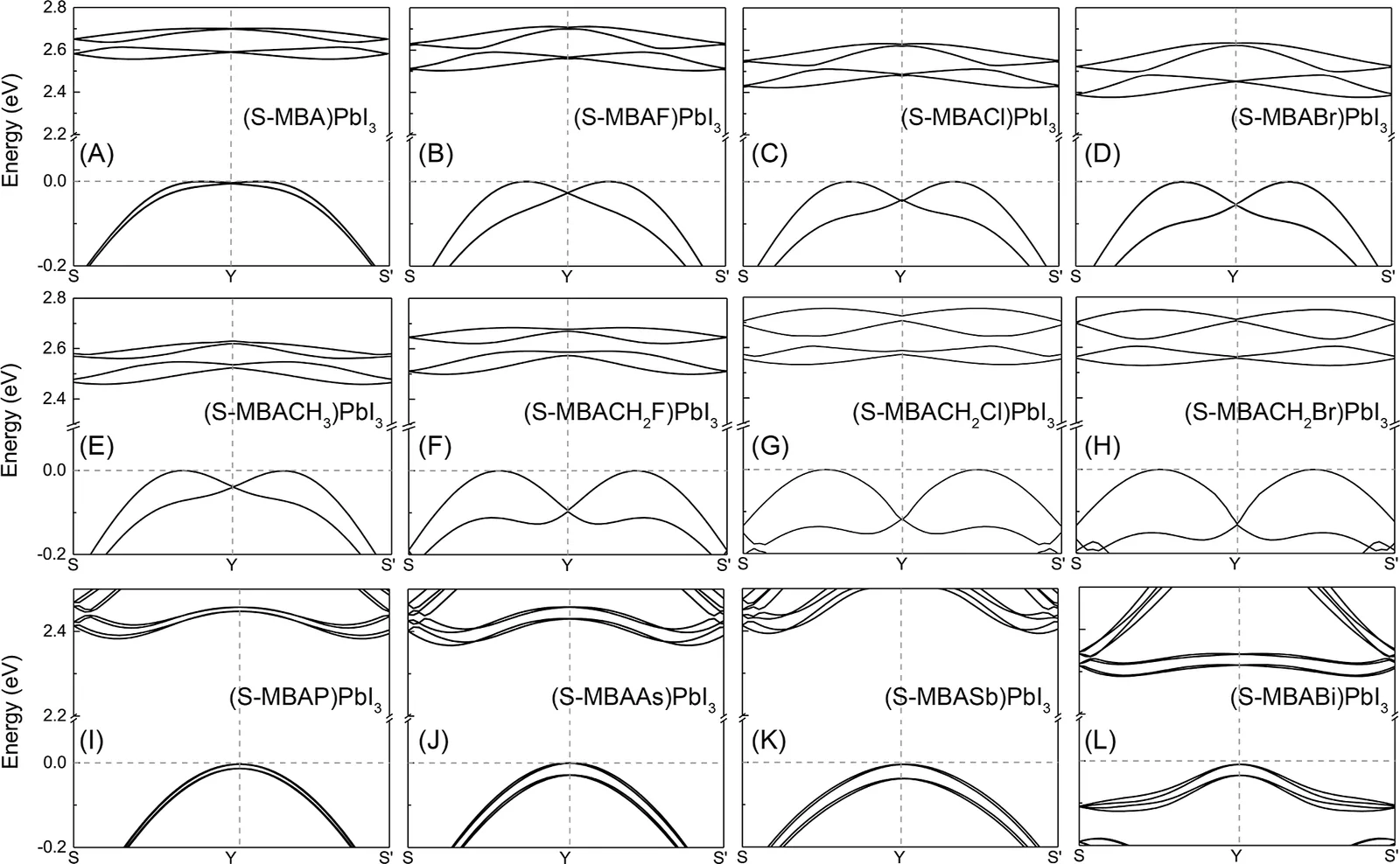
Band structures along the S–Y–S′ *k*-path for the three series studied here: (A–D) (S-MBA)­XPbI_3_; (E–H) (S-MBA)­CH_2_XPbI_3_; and (I–L) (S-MBA)­YPbI_3_. X = H, F, Cl, Br; Y = P, As, Sb, Bi. The VBM is set to 0 eV.

Broken inversion symmetry in these chiral HOIPs leads to SOC-induced spin splitting away from TRIM. We quantify the resulting energy scale using the conventional Rashba-type parameters *E*_R_, *k*_0_, and α_R_, but only as a directional parametrization along the selected S–Y–S′ path. Because *P*2_1_2_1_2_1_ is chiral and nonpolar, this splitting should not be interpreted as a canonical polar Rashba effect described by a single polar axis. Figure 4A shows the orthorhombic Brillouin zone for the (S-MBA)Br system. We extract the momentum offset and the energy scale of the splitting at the VBM near the TRIM point Y, as sketched in Figure 4B.

**Figure 4:**
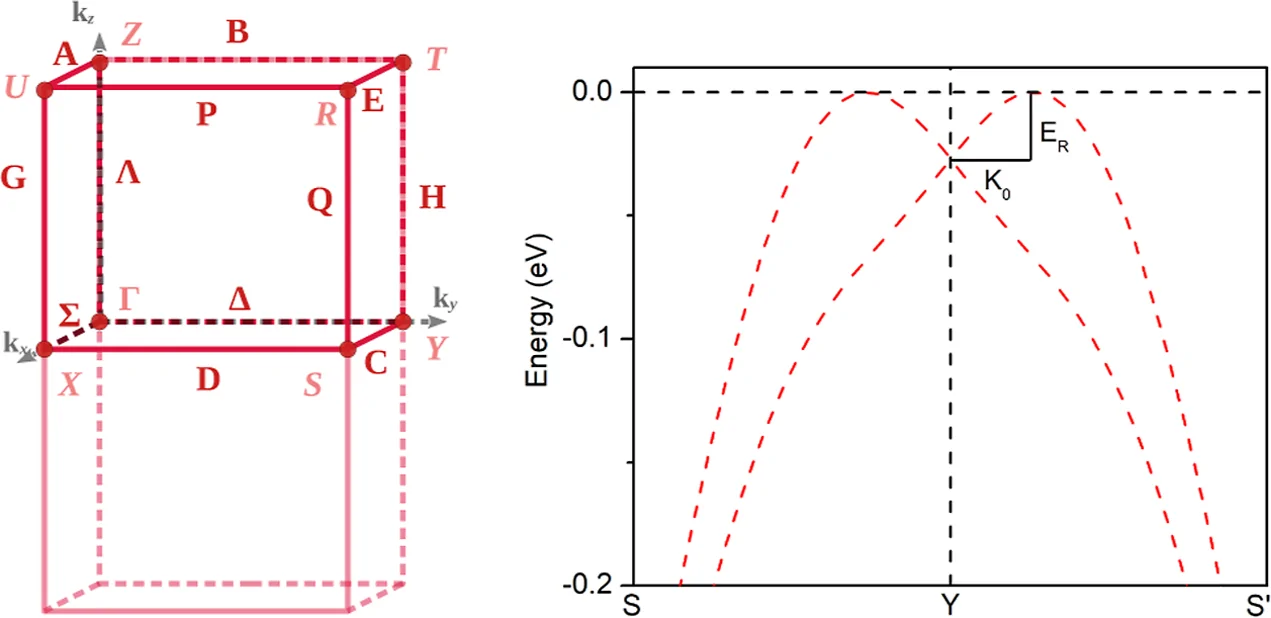
(Left) Brillouin zone of (S-MBA)­Br, highlighting the S–Y–S′ path. (Right) Top valence band of (S-MBA)Br along S–Y–S′, illustrating Rashba-type splitting near the TRIM point Y: the branches are Kramers-degenerate at Y, while the VBM extrema occur at ±*k*_0_. The Rashba energy *E*_R_ (from Y to the extremum) and *k*_0_ are used to compute α_R_ = 2*E*_R_/*k*_0_.

The momentum offset is denoted *k*_0_ (equivalently δ*k*). The corresponding energy scale *E*_R_ (equivalently δ*E*) is the Rashba energy measured between the Kramers-degenerate point at Y and the band extremum at ±*k*_0_; this definition differs from the energy separation between split branches at fixed **k**. The Rashba coefficient α_R_ is obtained from the effective two-band description as

2 $$\alpha_{R}=\frac{2E_{R}}{k_{0}}$$

The values of *E*_R_ and *k*_0_ were obtained by fitting the top valence band in a narrow **k**-window around Y along S–Y–S′. The computed parameters are listed in Table 1 and are consistent with previously reported results.^62^

**Table 1:** Rashba Parameters Extracted from the Top Valence Band for the (S-MBA)­X, (S-MBA)­CH_2_X, and (S-MBA)­Y Series (X = H, F, Cl, Br; Y = P, As, Sb, Bi)

series	*E*_R_ [meV]	*k*_0_ [Å^–1^]	α_R_ [eVÅ]
(S-MBA)	6.630	0.076	0.174
(S-MBA)F	26.871	0.112	0.480
(S-MBA)Cl	53.602	0.145	0.739
(S-MBA)Br	64.785	0.150	0.864
(S-MBA)CH_3_	36.562	0.135	0.542
(S-MBA)CH_2_F	95.005	0.165	1.152
(S-MBA)CH_2_Cl	109.970	0.187	1.176
(S-MBA)CH_2_Br	79.454	0.184	0.864
(S-MBA)P	1.500	0.005	0.600
(S-MBA)As	4.000	0.011	0.727
(S-MBA)Sb	5.000	0.010	1.000
(S-MBA)Bi	8.020	0.014	1.146

Several experimental studies are also closely related to the present work. Halogen substitution in HOIPs has been shown to modify lattice symmetry, increase structural distortion, and enhance the chiroptical response.^61^ Guan et al.^73^ further demonstrated that substituting uncoordinated halide ions improves structural distortion and phase stability while strongly enhancing both the linear and nonlinear chiroptical response, reporting an SHG-CD anisotropy factor of 1.56 and a luminescence quantum yield of 81.4%. Kwon et al.^74^ found that π-extended thiophene ligands with larger halogen substitutions increase lattice distortion and hydrogen bonding in 2D chiral perovskites, resulting in enhanced circular dichroism and luminescence. Jana et al.^75^ further showed that interactions between the organic spacer cations and the inorganic framework govern structural distortions, identifying descriptors such as octahedral tilting and bond angles that influence optoelectronic and spin-related properties. In this context, the present study provides a descriptor-based framework linking molecular substitution, chirality transfer, and spin splitting to guide future spectroscopic and spin-transport experiments.

Across all series, the valence band displays Rashba-type splitting near the TRIM point Y. In pristine (S-MBA)­PbI_3_, the splitting energy scale at the VBM is about 6 meV, consistent with ref 62. This value is below room-temperature thermal energy (*k*_B_ *T* ≈ 26 meV), motivating strategies to enhance the splitting for high-temperature spin-dependent applications.

Chirality is introduced through the organic cations; targeted ligand modification thus provides a direct route to tune spin splitting while retaining the parent Pb/I framework. Guided by experiments showing that halogen substitution enhances CD signals in related chiral perovskites,^61^ we introduce the halogenated (S-MBA)­X and (S-MBA)­CH_2_X series and the pnictogen-substituted (S-MBA)Y series.

Figure 3 shows that ligand substitution substantially modifies the valence-band dispersion near Y. In the (S-MBA)­X series, replacing H with halogens strongly enhances the spin splitting energies: *E*_R_ ≈ 26, 53, and 64 meV for F, Cl, and Br, respectively, compared with ∼6 meV in pristine (S-MBA)­PbI_3_. Halogen substitution also shifts the fundamental band gap *E*_g_ by about (0.1–0.2) eV (Table S4), providing an additional handle for band-structure engineering. In the (S-MBA)­CH_2_X series, the splitting increases from F to Cl but decreases for Br, indicating a competition between electronic effects and steric constraints. The (S-MBA)Y series instead yields *E*_R_ values in the few-meV regime, consistent with weaker CTE.

In all three substitution series, the VBM and CBM remain predominantly composed of Pb and I orbitals. Consequently, the effective SOC acting on the band-edge states is dominated by the common heavy-element inorganic framework and is expected to vary only weakly with ligand substitution. The pronounced variation of *E*_R_ across the series therefore cannot be attributed primarily to changes in the intrinsic SOC strength at the band edge; rather, the substitutions mainly modify the inversion-asymmetric crystal field and the efficiency of chirality transfer to the inorganic framework. This interpretation is supported by the pnictogen-substituted series as an internal control: replacing P with progressively heavier pnictogen substituents does not produce a comparable increase in *E*_R_, which remains in the few-meV range because the induced chiral distortion of the Pb/I framework is strongly suppressed. Thus, simply introducing heavier molecular substituents is insufficient to generate large band-edge spin splitting when the inorganic framework does not acquire the appropriate chiral distortion. Therefore, while SOC provided by the common Pb/I-derived band-edge states is a necessary prerequisite for spin splitting, the observed variations in *E*_R_ are governed by differences in inversion asymmetry and the efficiency of chirality transfer to the inorganic framework.

In the (S-MBA)­X series, α_R_ increases monotonically from H to Br, consistent with a progressively stronger inversion-asymmetric local field induced by ligand substitution. In the (S-MBA)­CH_2_X series, α_R_ increases from F to Cl, while the Br derivative shows a reduced α_R_, consistent with steric reorganization and dipole-driven reweighting of the local inversion-asymmetric field at the band edge. In the pnictogen-substituted (S-MBA)Y series, *E*_R_ remains in the few-meV regime; the moderate α_R_ values mainly reflect the small extracted *k*_0_ and should not be interpreted as evidence of large spin splitting energy.

### Impact of Structural Distortions on SOC-Induced Band-Edge Splitting

The wide range of computed splitting values observed across compounds sharing the same heavy-element inorganic component (Pb/I) indicates that SOC alone cannot account for the variability, highlighting the role of local structural distortions.^76^

For interoctahedral distortions, the steric and electrostatic constraints imposed by the organic cation dictate how the framework accommodates the molecular sublattice,^77,78^ including relative rotations, bending, and shear-like distortions of neighboring PbI_6_ units. We quantify this structural response using the three interoctahedral Pb–I–Pb angles, β_*i*_ (*i* = 1, 2, 3; Figure S1). These angles serve as local descriptors of linkage bending and angular anisotropy, rather than as conventional octahedral tilt angles of an ideal corner-sharing perovskite network.

The average linkage geometry is quantified by

3 $$\beta^{\prime }=\frac{1}{3}\sum_{i=1}^{3}\beta_{i}$$

whereas the anisotropy among the three linkages is measured by

4 $$\delta \beta =\frac{1}{3}\overset{3}{\underset{i=1}{\sum }}{\left(\beta_{i}-\beta^{\prime }\right)}^{2}$$

thus, β′ and δ β provide compact descriptors for the mean Pb–I–Pb linkage geometry and its angular dispersion, respectively. They complement the intraoctahedral distortion parameters Δ*d* and σ^2^, and help distinguish average framework bending from anisotropic linkage inequivalence.

Intraoctahedral distortion, on the other hand, is quantified by two geometric metrics: the bond length distortion and the bond-angle variance. The former is defined as

5 $$\Delta d=\frac{1}{6}\sum_{i=1}^{6}\frac{{\left(d_{i}-d_{0}\right)}^{2}}{d_{0}^{2}}$$

where *d*_*i*_ denotes the six M-X bond lengths and *d*_0_ is their mean value. This parameter captures deviations from uniform bond lengths, with larger values indicating stronger axial or equatorial elongation/compression within the octahedron. The bond-angle variance

6 $$\sigma^{2}=\frac{1}{11}\sum_{i=1}^{12}{\left(\theta_{i}-90\right)}^{2}$$

where θ_*i*_ denotes the individual cis X–M–X bond angles, quantifies deviations from the ideal cis bond angle of 90 deg.^79^ An undistorted MX_6_ octahedron yields Δ*d* = 0 and σ^2^ = 0, while larger values of each metric reflect stronger octahedral distortions.

Following ref 78, we examine how local octahedral distortion and Pb–I–Pb linkage geometry relate to the Rashba-type band-edge spin splitting observed in the valence band. Table 2 shows that framework distortion contributes to setting the magnitude of *E*_R_, but no single geometric descriptor provides a universal ranking across all three substitution series, in accordance with recent findings^80^ on the charge displacement of the octahedron for HOIPs. In the (S-MBA)­X series, *E*_R_ increases as the Pb–I–Pb linkage-angle anisotropy grows. However, fluorination yields a smaller Δ*d* than the parent compound despite having a much larger *E*_R_. In the (S-MBA)­CH_2_X series, all distortion metrics increase from CH_3_ to CH_2_Br, but *E*_R_ reaches its maximum at CH_2_Cl and then decreases for CH_2_Br. The pnictogen-substituted series provides a further counter example: As has smaller Δ*d* and σ^2^ than P yet a larger *E*_R_. Even the Bi derivative, the most distorted member of this series, remains far less distorted than the high-*E*_R_ halogenated systems and stays in the few-meV regime. Large *E*_R_ emerges only when bond-length inequivalence, Pb–I–Pb linkage-angle anisotropy, efficient amplification of the induced chiral Γ_1_^–^ mode, and a favorable electrostatic environment from the cation act cooperatively.

**Table 2:** Structural Parameters for the (S-MBA)­X, (S-MBA)­CH_2_X, and (S-MBA)Y Series (X = H, F, Cl, Br; Y = P, As, Sb, Bi)^a^

series	**Δ** *d*[10^–5^]	**σ** ^**2**^[◦^2^]	**β**_**1**_[◦]	**β**_**2**_[◦]	**β**_**3**_[◦]	**β′**[◦]	**δ β**[◦^2^]
(S-MBA)	61.51	67.62	76.32	76.63	77.20	76.71	0.13
(S-MBA)F	52.90	93.78	75.29	74.54	74.07	74.63	0.25
(S-MBA)Cl	105.62	74.64	72.08	73.74	74.45	73.42	0.98
(S-MBA)Br	122.01	73.85	71.53	73.48	73.97	72.99	1.11
(S-MBA)CH_3_	62.45	73.11	72.66	74.32	74.51	73.84	0.69
(S-MBA)CH_2_F	99.28	116.32	74.52	76.26	76.55	75.78	0.80
(S-MBA)CH_2_Cl	361.80	130.71	74.04	78.84	78.74	77.20	5.01
(S-MBA)CH_2_Br	553.83	142.37	72.87	79.67	79.59	77.38	10.15
(S-MBA)P	3.91	11.97	72.08	72.81	72.90	72.59	0.13
(S-MBA)As	2.44	5.32	72.05	72.38	72.82	72.42	0.10
(S-MBA)Sb	5.45	5.36	72.10	73.09	72.89	72.70	0.18
(S-MBA)Bi	15.90	8.28	73.27	71.43	73.63	72.78	0.92

a Here β_*i*_ are the three Pb–I–Pb linkage angles, β′ = (1/3)∑_*i*_β_*i*_, and $\delta \beta =\left(1/3\right)\sum_{i}{\left(\beta_{i}-\beta^{\prime }\right)}^{2}$ .

Notably, the (S-MBA)Y series exhibits relatively small mean Pb–I–Pb angles β′ yet remains in the few-meV regime. This indicates that average framework bending alone is insufficient to generate large band-edge spin splitting without strong linkage anisotropy and efficient chirality transfer to the Pb/I framework.

### Electronic Chirality, Parity-Violating Energy, and Cation Dipoles

To rationalize the Rashba-type band-edge spin splitting, we examine how molecular descriptors—ECM, PV energy contributions, and the intrinsic electric dipole moment—computed for the isolated organic cations extracted from the relaxed crystal structures vary across the halogenated and pnictogen-substituted cation series.

We summarize the results for the ECM, PV contributions, and the intrinsic electric dipole moment in Table 3.

**Table 3:** ECM, Δ*E* ^PV^ [a.u.] and Intrinsic Electric Dipole Magnitude |p| [a.u.] for the Isolated Organic Cations Extracted from the Relaxed Crystal Structures

series	ECM	Δ*E* ^PV^ [a.u.]	|p| [a.u.]
(S-MBA)	19.60	1.73 × 10^–21^	3.15
(S-MBA)F	30.11	2.41 × 10^–20^	2.81
(S-MBA)Cl	37.56	8.70 × 10^–20^	2.59
(S-MBA)Br	48.64	8.59 × 10^–19^	2.59
(S-MBA)CH_3_	17.48	1.24 × 10^–21^	2.77
(S-MBA)CH_2_F	27.95	1.14 × 10^–20^	2.04
(S-MBA)CH_2_Cl	32.33	6.29 × 10^–20^	1.91
(S-MBA)CH_2_Br	42.67	6.37 × 10^–19^	2.14
(S-MBA)P	18.68	1.07 × 10^–20^	2.75
(S-MBA)As	22.14	5.39 × 10^–19^	1.90
(S-MBA)Sb	19.68	2.20 × 10^–17^	1.46
(S-MBA)Bi	12.54	7.98 × 10^–15^	0.41

For the (S-MBA)­X and (S-MBA)­CH_2_X series, Figure 5 (left) shows that Δ*E* ^PV^ and ECM exhibit qualitatively consistent trends.

**Figure 5:**
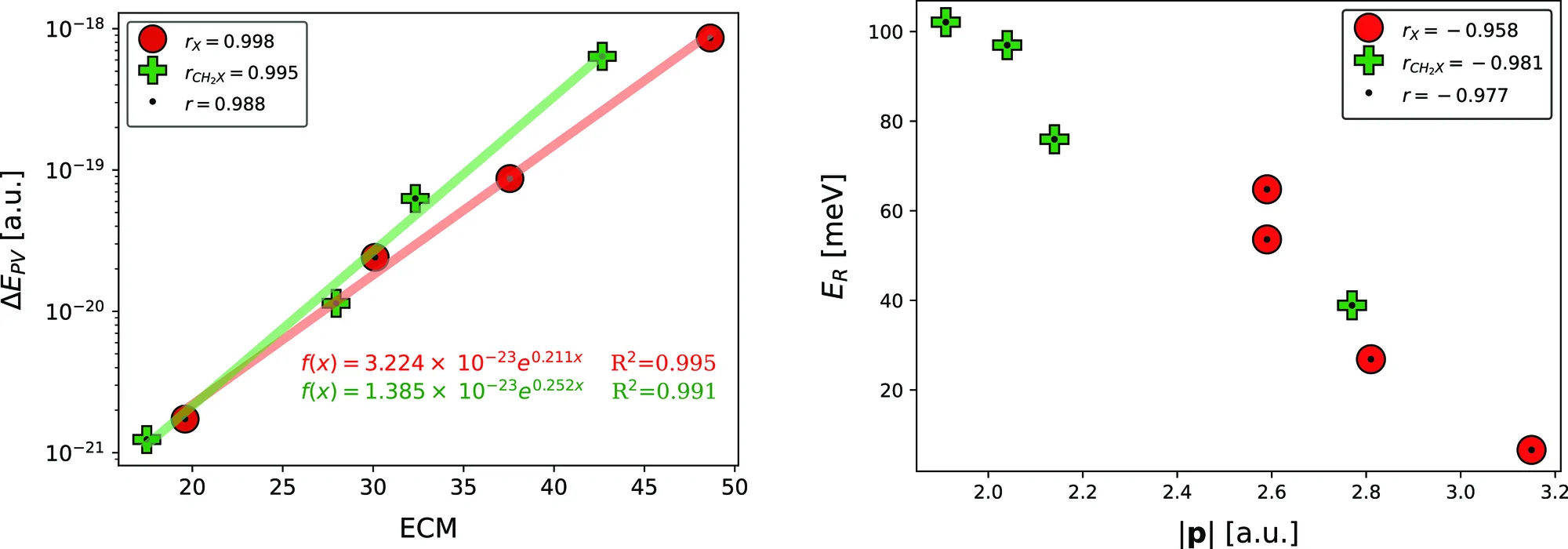
Results for the two halogenated series, (S-MBA)­X and (S-MBA)­CH_2_X (X = H, F, Cl, Br). The Pearson correlation coefficient *r* is reported. (Left) scatter plot for Δ*E* ^PV^ [a.u.] versus ECM. Pearson coefficients are computed for ln­(Δ*E* ^PV^) versus ECM, and the exponential fits are shown as descriptive guides to the eye. (Right) correlation between the Rashba-type band-edge spin splitting energy scale *E*_R_ [meV] and the intrinsic cation dipole magnitude |**p**| [a.u.].

The Pearson coefficients computed for ln­(Δ*E* ^PV^) against ECM indicate a positive association for the first and second series, both when treated separately (*r*_X_ = 0.998, *n* = 4; $r_{CH_{2}X}=0.995$ , *n* = 4) and when combined (*r* = 0.988, *n* = 8). Given the limited sample size within each series (*n* = 4), we interpret these coefficients as descriptive of the present chemical space rather than as evidence of a robust statistical law. Their remarkable internal consistency nevertheless motivated the descriptor analysis developed here. The same caveat applies to the dipole correlations discussed below. The dependence of Δ*E*_PV_ on ECM is well described by an exponential form, confirming previous findings.^13^

Before discussing the dipole correlations, it is important to distinguish the physical origin of the quantities examined above. The parity-violating energy difference Δ*E* ^PV^ is a molecular property governed by the weak interaction and strongly enhanced by heavy-atom relativistic effects in the cation, and ECM originates from the chirality degree of the molecular cations. By contrast, the band-edge spin splitting *E*_R_ is a solid-state SOC phenomenon associated with Pb/I-derived band-edge states in a noncentrosymmetric crystal field. Therefore, ECM quantifies the molecular electronic chirality, whereas *E*_R_ measures a response of the inorganic band edge. Strong molecular chirality does not guarantee large band-edge splitting, because efficient propagation of chirality into the inorganic framework and a favorable local electrostatic environment at the Pb/I band edge are also required, as will be shown for the series under study.

We next examine whether the intrinsic cation dipole helps rationalize the Rashba energy scale of the corresponding HOIPs. A rather weak correlation between both quantities was previously found,^39^ with the Pearson coefficient (*r*) being 0.03 (0.50) for *V*_B_(*C*_B_) in Sb-containing systems and 0.16 (−0.21) for *V*_B_(*C*_B_) in Pb-containing systems. However, if the cation dipole is disentangled from the inorganic framework contribution, a moderate negative correlation is observed, with *r* being −0.77 (−0.59) for *V*_B_(*C*_B_) in Sb-containing systems and −0.18 (−0.43) in Pb-containing systems. This indicates that, in both cases, the spin splitting energy decreases as the cation dipole increases.

Figure 5 (right) compares *E*_R_ with the intrinsic dipole magnitude |**p**| for the two halogenated series. Although the organic cation does not contribute directly to the Pb/I-derived band-edge states, its electrostatic field modifies the local crystal field experienced by those states. Consequently, depending on its magnitude and equilibrium orientation, the intrinsic dipole can reinforce, reduce, or redirect the local inversion-asymmetric field. The dipole magnitude |**p**| is therefore employed here as a molecular descriptor of the local electrostatic environment rather than as a direct microscopic measure of the inversion-asymmetric field itself. In both cases, the Pearson coefficient exceeds 0.95 in absolute value (*r*_X_ = −0.958, *n* = 4; $r_{CH_{2}X}=-0.981$ , *n* = 4), indicating a strong negative correlation. Combining the first and second series yields *r* = −0.977 (*n* = 8). Thus, within each halogenated series, larger |**p**| values are associated with smaller *E*_R_, consistent with earlier indications that the cationic electrostatic contribution can oppose rather than reinforce the local inversion-asymmetric field.^39^

Qualitatively, the observed negative association is consistent with an orientation-dependent electrostatic remodulation of the local inversion-asymmetric crystal field produced by the organic cation. Because the intrinsic dipole magnitude |p| is a scalar quantity, it cannot by itself determine the direction or spatial distribution of the local electrostatic field experienced by the Pb/I-derived frontier states. Consequently, the present correlation should be interpreted as a descriptor–level association within the investigated substitution series, rather than as direct evidence of an electrostatic screening mechanism. A rigorous microscopic demonstration would require real-space analyses of the electrostatic potential and orbital distributions, which are beyond the scope of the present descriptor-based study.

Figure 6 shows that, in the pnictogen-substituted series, *E*_R_ remains in the few-meV range, much lower than in the halogen-functionalized systems, even though the derived α_R_ values can be moderate due to a small *k*_0_. This series also exhibits a strong negative association between *E*_R_ and |**p**|. By contrast, ECM varies only weakly across the (S-MBA)Y series and decreases markedly for the Bi derivative. This behavior is consistent with the substituted site lying close to a symmetry plane of the nearest achiral reference structure,^15^ which increases the overlap entering the ECM definition for orbitals with appreciable weight on the substituted atom.

**Figure 6:**
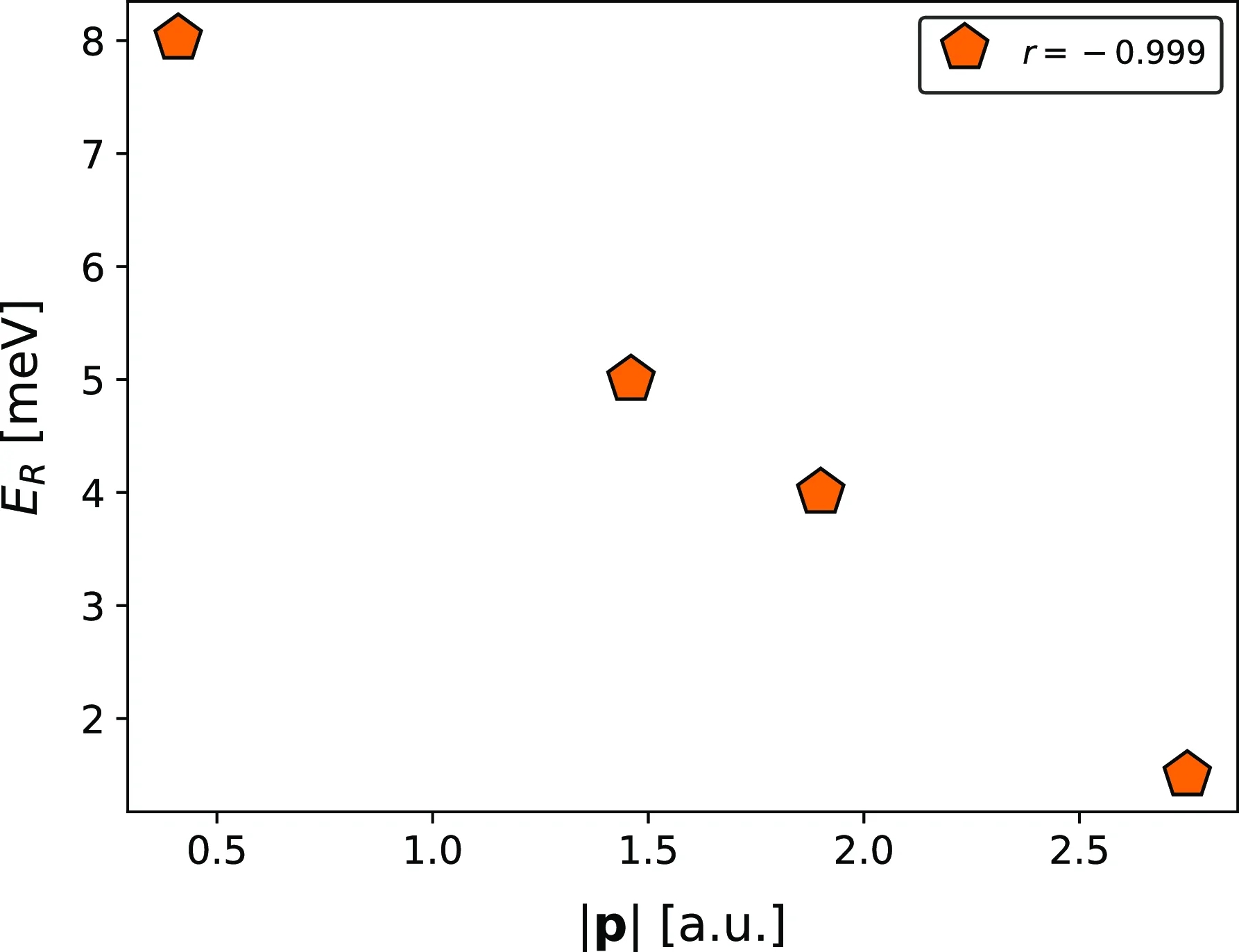
Correlation between the Rashba-type band-edge spin splitting energy scale *E*_R_ [meV] and the intrinsic cation dipole magnitude |**p**| [a.u.] for the (S-MBA)Y series (Y = P, As, Sb, Bi). The Pearson correlation coefficient *r* is reported.

Figure 7 summarizes the coupled molecular-framework descriptor space. Within the two halogenated series, *E*_R_ increases with $Q_{\Gamma_{1}^{-}}/Q_{glob}$ , demonstrating that amplification of the induced chiral framework mode is a key structural requirement for sizable band-edge splitting. The side-chain-functionalized derivatives occupy the high-*E*_R_ region, in which the induced chiral framework mode is efficiently amplified and the cation electrostatic environment is favorable, whereas the pnictogen-substituted systems remain clustered at very small $Q_{\Gamma_{1}^{-}}/Q_{glob}$ and low *E*_R_. These trends are observed for the present substitution series and indicate that CTE, rather than molecular chirality alone, is required to achieve large band-edge spin splitting. The observed association with the intrinsic cation dipole is therefore interpreted at the descriptor level and should not be taken as direct evidence of an electrostatic screening mechanism. Accordingly, the figure should be interpreted as a series-specific descriptor map summary rather than a universal scalar law.

**Figure 7:**
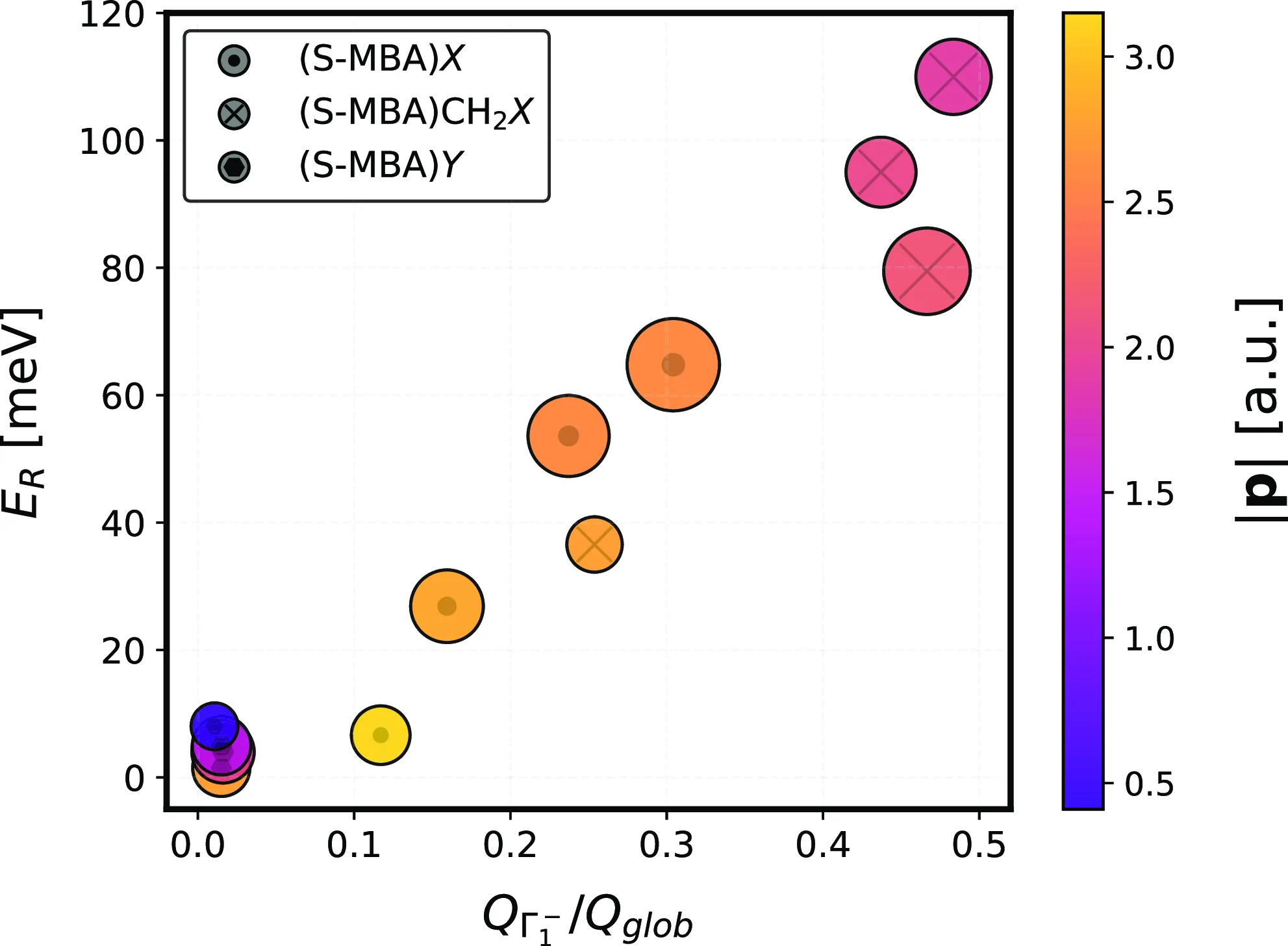
Integrated molecular–framework descriptor map for the three substitution series. The vertical axis reports the SOC-induced band-edge spin splitting energy scale *E*_R_ [meV], and the horizontal axis reports the framework chirality-transfer efficiency $Q_{\Gamma_{1}^{-}}/Q_{glob}$ . Marker shapes distinguish the (S-MBA)­X, (S-MBA)­CH_2_X, and (S-MBA)Y series (X = H, F, Cl, Br; Y = P, As, Sb, Bi); marker color encodes the intrinsic cation dipole magnitude |**p**| [a.u.]; and marker area is proportional to the ECM of the isolated organic cation.

## Conclusion

This study shows that band-edge spin splitting in chiral Pb/I hybrid perovskites is controlled by a coupled descriptor space rather than by molecular chirality alone. Halogen functionalization raises *E*_R_ from 6.6 meV in (S-MBA)­PbI_3_ to 64.8 meV in the (S-MBA)­X series and up to 110.0 meV in the (S-MBA)­CH_2_X series (X = H, F, Cl, Br), whereas pnictogen substitution leaves *E*_R_ in the few-meV regime for the (S-MBA)Y series (Y = P, As, Sb, Bi) despite strongly enhanced Δ*E* ^PV^. Framework-only symmetry-mode analysis identifies the induced Γ_1_^–^ mode and its normalized amplitude (CTE) as the clearest structural fingerprints of chirality transfer: the (S-MBA)­X series selectively amplifies it, the (S-MBA)­CH_2_X series amplifies it cooperatively with the primary distortion sector, and the (S-MBA)­Y series largely quenches it.

ECM provides a useful indicator of the changes in chiral electronic structure, but it does not uniquely determine the band-edge splitting, because an efficient propagation of the molecular chirality into the inorganic framework is required. Within each chemical series, larger intrinsic cation dipoles are associated with smaller *E*_R_, consistent with orientation-dependent screening or remodulation of the local inversion-asymmetric fields at the Pb/I band edges. Large spin splitting therefore requires efficient conversion of framework distortion into the induced chiral mode together with a favorable cation electrostatic environment. This identifies the CTE as a key structural descriptor linking molecular chirality to the inorganic framework response. These findings, established for the present substitution series rather than as universal statistical rules, provide a mechanistic basis for prioritizing molecular substitutions in chiral spin-optoelectronic hybrid perovskites.

The present analysis is based on the relaxed ordered crystal structures obtained from first-principles calculations. In real samples, orientational disorder of the organic cations, dynamic molecular motion, or the presence of opposite chiral domains may modify the local electrostatic environment experienced by the band-edge states. Such effects can reduce the experimentally observed effective spin splitting or spin-polarization signals through configurational averaging, although they do not alter the descriptor-based trends identified for the ideal ordered structures considered in this work.

## Supplementary Material



## Data Availability

The data that support the findings of this study are available from the corresponding authors upon reasonable request.

## Notes

Relaxed lattice parameters, symmetry-mode amplitudes, PBE + SOC band gaps, definitions of parity-violating energy and ECM, electric dipole-moment convention, SOC/*k* × *p* formalism, and additional computational details (PDF)
