# Supplementary material for: Coupled Molecular and Framework Descriptors for Band-Edge Spin Splitting in Chiral Hybrid Organic–Inorganic Perovskites
Source: J Am Chem Soc. 2026 Aug 26;148(35):38304–15. doi: 10.1021/jacs.6c11593 (PMC13564420; doi:10.1021/jacs.6c11593)
Supplement: Supplementary file 1 [file ja6c11593_si_001.pdf]

Supporting Information: Coupled Molecular and Framework Descriptors for Band-Edge Spin Splitting in Chiral Hybrid Organic–Inorganic Perovskites

Muskan Nabi,[†,‖] Juan J. Aucar,[‡,‖] Xinfeng Chen,[*,¶] Gustavo A. Aucar,[*,‡] and Alessandro Stroppa[*,§]

†*CNR-SPIN, Department of Physical and Chemical Sciences, University of L'Aquila, Via Vetoio, 67100 L'Aquila, Italy*

‡*Institute for Modelling and Innovative Technology, IMIT (CONICET-UNNE), Avda Libertad 5460, W3404AAS, Corrientes, Argentina*

¶*Key Lab of advanced optoelectronic quantum architecture and measurement (MOE), Beijing Key Laboratory of Quantum Matter State Control and Ultra-Precision Measurement Technology, and School of Physics, Beijing Institute of Technology, Beijing 100081, China*

§*CNR-SPIN, c/o Dip.to di Scienze Fisiche e Chimiche - Via Vetoio - 67100 - Coppito (AQ), Italy*

‖*These authors contributed equally to this work.*

E-mail: zkchen@bit.edu.cn; gaaucar@conicet.gov.ar; alessandro.stroppa@spin.cnr.it

Here we provide supplementary structural, symmetry-mode, and methodological details for the three series studied in this work: the benzylic-halogenated (S-MBA)X series, the side-chain-halogenated (S-MBA)CH_2X series and the pnictogen-substituted (S-MBA)Y series (X = H, F, Cl, Br ; Y = P, As, Sb, Bi). Table S1 lists the relaxed lattice parameters and unit-cell volumes. The framework-only symmetry-mode amplitudes discussed in the main text are reported in Tables S2–S3. In the section on spin–orbit coupling and the spin splitting effects, Table S4 presents the PBE+SOC band gaps for all three substitution series. The following sections then provide the theoretical details on parity-violating energy, the electronic chirality measure, spin–orbit coupling, the electric dipole moment, and the relativistic origin of the Rashba-type Hamiltonian.

Structural details

All optimized structures retain the orthorhombic chiral space group $P2_12_12_1$ (No. 19). Table S1 lists the relaxed lattice parameters and unit-cell volumes. The three substitution series display distinct lattice responses already at the level of the full crystal: the (S-MBA)X series shows an overall contraction relative to the parent compound, the (S-MBA)CH_2X series develops the largest changes in volume and anisotropy, and the (S-MBA)Y series mainly expands the lattice along the c axis while preserving a comparatively regular PbI_6 framework. A schematic definition of the interoctahedral Pb–I–Pb angles β_i is shown in Figure S1.

Framework-Only Structural Analysis

To isolate the inorganic contribution to chirality transfer, the organic molecules were removed and the relaxed Pb/I frameworks were analyzed with the Bilbao `Structure Relations`/`PSEUDO` tools[S1] and `AMPLIMODES`.[S2] The resulting framework-only structures were compared with symmetrized *Imma* parent structures. For intercompound comparisons, we retain only the amplitudes normalized with respect to the primitive unit cell of the high-symmetry structure,

Table S1: Relaxed lattice parameters and unit-cell volumes for the (S-MBA)X, (S-MBA)CH_2X and (S-MBA)Y series (X = H, F, Cl, Br ; Y = P, As, Sb, Bi).

Compound	a [Å]	b [Å]	c [Å]	V [Å^3]
(S-MBA)	8.0950	8.7141	21.3680	1507.3
(S-MBA)F	7.8560	8.7000	20.9572	1432.4
(S-MBA)Cl	7.8093	9.1380	21.0157	1499.7
(S-MBA)Br	7.7456	9.1138	20.7744	1466.5
(S-MBA)CH_3	7.8132	9.1424	20.5582	1468.5
(S-MBA)CH_2F	7.9567	9.0087	20.2866	1454.1
(S-MBA)CH_2Cl	8.1791	9.1082	20.3779	1518.1
(S-MBA)CH_2Br	8.2433	9.1380	20.5295	1546.4
(S-MBA)P	7.6635	8.7643	21.3986	1437.2
(S-MBA)As	7.6762	8.7872	22.5467	1520.8
(S-MBA)Sb	7.6806	8.8354	21.8864	1485.2
(S-MBA)Bi	7.7166	9.0158	23.1398	1609.9

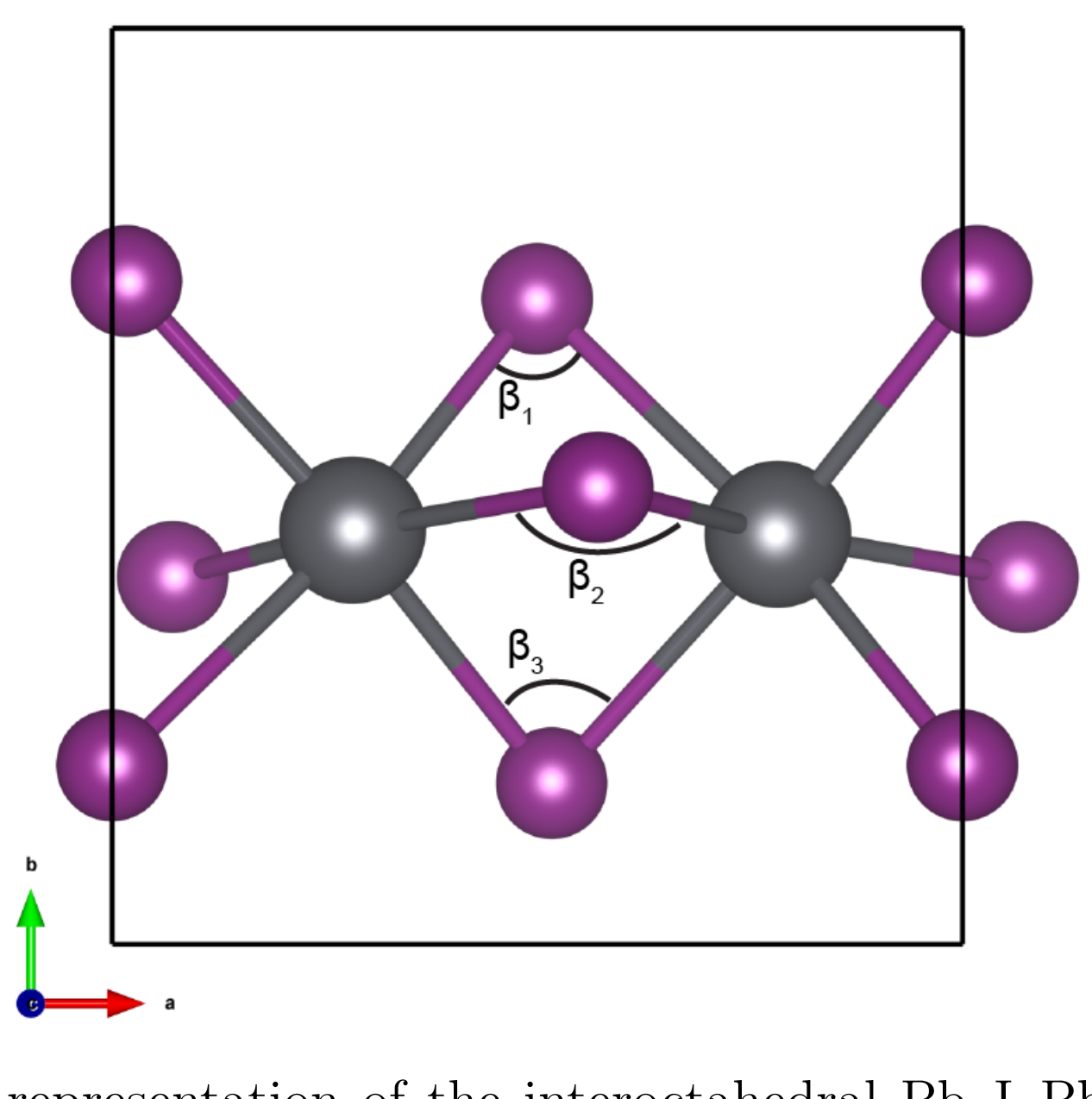

Figure S1: Schematic representation of the interoctahedral Pb–I–Pb bond angles β_i. The organic molecules are omitted for clarity.

because the alternative normalization supplied by the Bilbao output is not directly comparable across structures with different primitive-cell multiplications. The reported $Q_{X_4^+}$, $Q_{X_4^-}$, $Q_{\Gamma_1^-}$ and Q_{Global} values therefore correspond throughout the SI to the high-symmetry-cell normalization.

Across the three series, the same symmetry-lowering pattern is recovered for the isolated Pb/I framework: two dominant achiral zone-boundary modes, X_4^+ and X_4^-, a negligible Γ_1^+ contribution, and a finite chiral zone-center mode Γ_1^-. The corresponding amplitudes are collected in Table S2. This common mode content is consistent with a trilinear coupling term

$$F_{\mathrm{tri}} = \lambda Q_{X_4^+} Q_{X_4^-} Q_{\Gamma_1^-},$$

in which the chiral framework mode is induced as a secondary order parameter by two primary achiral distortions. The induced Γ_1^- mode breaks inversion symmetry and is primarily encoded as a handed shear of symmetry-related equatorial iodides, with only a small Pb counter-motion. The X_4^+ and X_4^- modes are the primary achiral distortions that provide the symmetry-breaking channel. Their presence is necessary but not sufficient for efficient chirality transfer, as illustrated by the pnictogen-substituted series, where the primary achiral response remains finite while conversion into the chiral mode is weak because the substitution couples less efficiently to the iodide sublattice.

Microscopic Character of the Induced Chiral Mode

The induced Γ_1^- mode is the clearest structural fingerprint of chirality transfer. In the two halogenated series, (S-MBA)X and (S-MBA)CH_2X, the corresponding normalized polarization vector is overwhelmingly iodide-dominated: the I contribution remains close to unity, whereas the Pb contribution stays small (Table S3). Structurally, this means that chirality transfer is encoded mainly as a handed shear of the equatorial iodide sublattice, accompanied by only a small Pb counter-displacement. The (S-MBA)X series selectively amplifies

Table S2: Framework-only symmetry-mode amplitudes (in Å) obtained from `AMPLIMODES`, normalized with respect to the primitive unit cell of the high-symmetry structure. Q_{glob} denotes the global distortion amplitude.

Compound	$Q_{\Gamma_1^+}$	$Q_{\Gamma_1^-}$	$Q_{X_4^+}$	$Q_{X_4^-}$	Q_{glob}
(S-MBA)	0.0002	0.1416	0.9317	0.7598	1.2105
(S-MBA)F	0.0002	0.2249	1.0801	0.8793	1.4109
(S-MBA)Cl	0.0002	0.2617	0.9102	0.5659	1.1033
(S-MBA)Br	0.0002	0.3100	0.8064	0.5405	1.0191
(S-MBA)CH_3	0.0000	0.2504	0.8293	0.4721	0.9866
(S-MBA)CH_2F	0.0000	0.5026	0.9207	0.4711	1.1499
(S-MBA)CH_2Cl	0.0002	0.6731	1.1155	0.4908	1.3922
(S-MBA)CH_2Br	0.0002	0.7612	1.2973	0.6321	1.6316
(S-MBA)P	0.0002	0.0195	1.2922	0.1700	1.3034
(S-MBA)As	0.0001	0.0276	1.7067	0.1497	1.7135
(S-MBA)Sb	0.0002	0.0239	1.5663	0.1536	1.5740
(S-MBA)Bi	0.0000	0.0232	2.1627	0.2471	2.1769

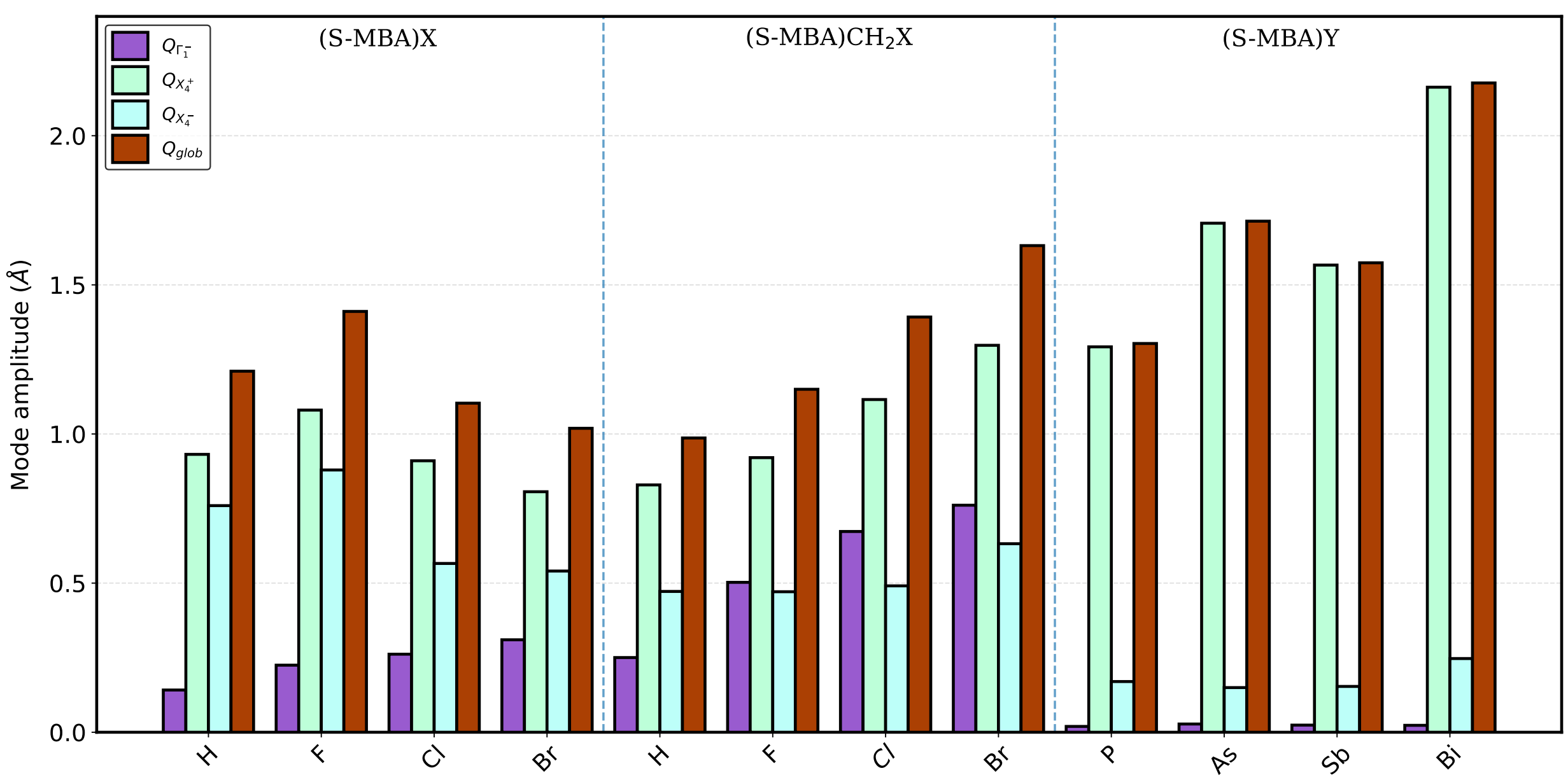

Figure S2: Framework-only symmetry-mode amplitudes across the (S-MBA)X, (S-MBA)CH_2X and (S-MBA)Y series (X = H, F, Cl, Br ; Y = P, As, Sb, Bi). Grouped bars report $Q_{\Gamma_1^-}$, $Q_{X_4^+}$, $Q_{X_4^-}$ and Q_{glob}, using the normalization with respect to the primitive unit cell of the high-symmetry structure.

this chiral mode, because $Q_{\Gamma_1^-}$ increases from the parent to the F, Cl, and Br derivatives even though the primary $X_4^{\pm}$ sector and the global distortion amplitude are nonmonotonic (Table S2).

The (S-MBA)CH_2X series enters a more cooperative regime. From CH_3 to CH_2Br, $Q_{\Gamma_1^-}$, $Q_{X_4^+}$, and Q_{glob} all increase, showing that the primary achiral distortions and the induced chiral mode are amplified together. In the (S-MBA)Y series, the same trilinear pattern remains symmetry-allowed, but the coupling becomes strongly unbalanced: $Q_{X_4^+}$ remains sizeable and even increases toward the heavier pnictogens, whereas $Q_{\Gamma_1^-}$ is strongly suppressed. The corresponding Γ_1^- eigenvector also becomes less uniformly iodide-dominated, especially for P, Sb, and Bi where the Pb contribution is comparatively larger (Table S3). The pnictogen-substituted (S-MBA)Y series therefore demonstrates that sizeable framework distortion by itself is not sufficient to generate an efficient chiral distortion of the Pb/I network.

Table S3: Dominant coefficients of the normalized polarization vector for the induced Γ_1^- mode. The reported values correspond to the I and Pb contributions provided by `AMPLIMODES`. The sign retains the phase convention of the Bilbao mode vectors; the relative magnitude reflects whether the induced chiral mode is predominantly iodide- or Pb-driven.

Compound	I coefficient in Γ_1^-	Pb coefficient in Γ_1^-
(S-MBA)	0.9801	0.1985
(S-MBA)F	0.9911	-0.1330
(S-MBA)Cl	0.9927	0.1210
(S-MBA)Br	0.9913	0.1314
(S-MBA)CH_3	0.9983	-0.0589
(S-MBA)CH_2F	0.9600	-0.2801
(S-MBA)CH_2Cl	0.9946	-0.1039
(S-MBA)CH_2Br	0.9969	-0.0786
(S-MBA)P	0.5230	-0.8523
(S-MBA)As	0.9902	-0.1396
(S-MBA)Sb	0.0738	-0.9973
(S-MBA)Bi	0.5959	-0.8031

Band gaps

The PBE+SOC band gaps of the (S-MBA)X, (S-MBA)CH_2X, and (S-MBA)Y series are summarized in Table S4.

Table S4: Calculated band gaps (in eV) for the (S-MBA)X, (S-MBA)CH_2X and (S-MBA)Y series (X = H, F, Cl, Br ; Y = P, As, Sb, Bi) using the PBE+SOC approximation.

Series	E_g [eV]
(S-MBA)	2.55
(S-MBA)F	2.41
(S-MBA)Cl	2.40
(S-MBA)Br	2.37
(S-MBA)CH_3	2.45
(S-MBA)CH_2F	2.49
(S-MBA)CH_2Cl	2.53
(S-MBA)CH_2Br	2.52
(S-MBA)P	2.38
(S-MBA)As	2.36
(S-MBA)Sb	2.29
(S-MBA)Bi	2.39

Parity-Violating Energy

In molecules, the parity-violating (PV) interactions between electrons and nuclei arise primarily from the lowest-order Z^0 boson exchange. In the relativistic regime, these interactions are predominantly captured by the nuclear spin-independent mechanism, which can be expressed through the effective Hamiltonian[S3,S4]

$$\hat{H}^{\mathrm{PV}}_{\mathrm{eff}} = \frac{G_F}{2\sqrt{2}} \sum_{i,A} Q_{W,A} \gamma^5_i \varrho_A(\mathbf{r}_i) \tag{S.1}$$

where the nucleons inside the nucleus are treated as nonrelativistic.[S5] The indices i and A run over all electrons and nuclei, respectively. G_F denotes the Fermi coupling constant, $G_F \approx 2.222516 \times 10^{-14} a.u.$[S6] The weak nuclear charge is defined as $Q_{W,A} = Z_A(1 - 4\sin^2\theta_W) - N_A$, where Z_A and N_A are the number of protons and neutrons of nucleus A, respectively. For

$sin^2\theta_W$ we use 0.23121(4).[S7] The Dirac matrices γ_i^5 and $\boldsymbol{\alpha}_i$ act on the spinor of electron i; $\mathbf{r}_i$ denotes the electron position relative to the coordinate origin, and $\varrho_A(\mathbf{r}_i)$ is the normalized nucleon density distribution of nucleus A at the position of electron i, related to the nuclear charge density via $\varrho_A(\boldsymbol{r}_i) = \rho_A(\boldsymbol{r}_i)/(Z_A e)$. Treating the PV effective Hamiltonian as a perturbation to the Dirac-Coulomb Hamiltonian, the first-order contribution to the electronic energy, E^{PV}, is obtained as the expectation value of Eq. (S.1) over the solutions of the Dirac–Coulomb equation. This contribution vanishes for achiral molecules, while for the two enantiomers of a chiral molecule it produces nonzero energies of equal magnitude but opposite sign,[S8] resulting in a ground-state energy difference expressed as

$$\Delta E^{\mathrm{PV}} = 2\left|E^{\mathrm{PV}}\right| = \left|E_R^{PV} - E_S^{PV}\right| \tag{S.2}$$

As in Ref. S9, we confirm the dependence of ΔE^{PV} on the atomic number Z_A of the nucleus under substitution. For the two halogenated series, (S-MBA)X and (S-MBA)CH_2X, it is observed in Fig. S4 (top) that ΔE^{PV} increases as a power law with the substituent atomic number Z_A, with an effective exponent close to ~ 3 over the present chemical range, consistent with recent findings[S10,S11] indicating that subtle electronic-structure effects, rather than exclusively a Z_A^5 scaling law,[S8,S12–S14] can be the cause of the strong enhancement in the PV contributions to molecular energy for chiral molecules. The H reference points are not included in the Z-scaling fits, because H is not a heavy substituent in the sense relevant to PV enhancement. It should be noted that ΔE_{PV} is not invoked in this manuscript as a direct energetic contribution to the SOC-induced band splitting. Rather, it is used as a relativistic molecular descriptor that tracks changes in chiral electronic structure and heavy-atom response within the isolated organic cations.

In Fig. S3 the representative molecular structures of the three series under substitution are shown. Fig. S4 (bottom) shows that the (S-MBA)Y series follows a strong exponential relationship between ΔE^{PV} and the atomic number of the substituted atom (R^2=0.998),

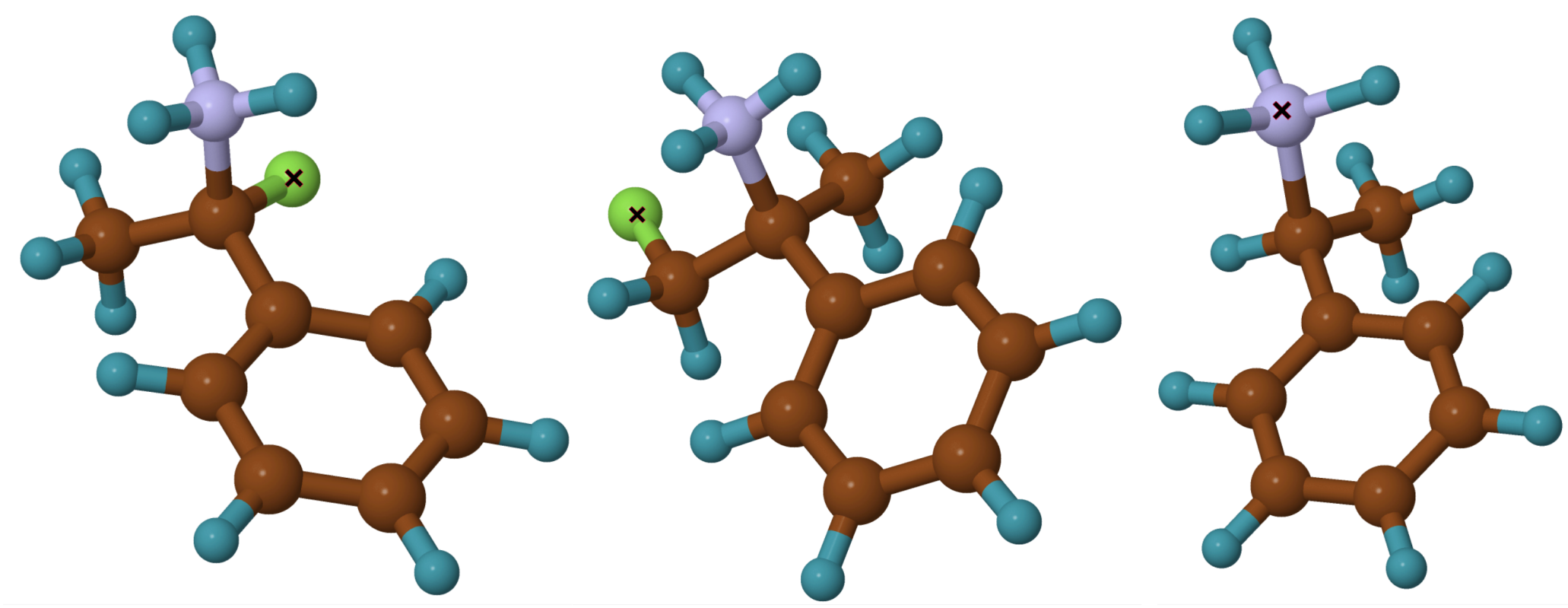

Figure S3: Representative molecular structures of the three substitution series: the benzylic-halogenated (S-MBA)F cation (left), the side-chain-halogenated (S-MBA)CH_2F cation (center), and the pnictogen-substituted (S-MBA)P cation (right). These molecules illustrate the (S-MBA)X, (S-MBA)CH_2X, and (S-MBA)Y series, respectively (X = H, F, Cl, Br ; Y = P, As, Sb, Bi).

leading to a substantial increase in PV effects. Because Sb and Bi are much heavier than Br, ΔE^{PV} increases by several orders of magnitude.

Electronic Chirality Measure

The Electronic Chirality Measure (ECM) descriptor provides an *electronic* quantification of the chirality for any molecular system. The method is based on defining the operator $\hat{S} = 1 - \hat{Z}$, where $\hat{Z}$ represents the operation that maps the wave function centered on the original atomic positions to the corresponding wave function centered on the atoms in the nearest symmetric achiral configuration of the molecule.[S15] The two wave functions are defined for different geometric configurations. The full set of molecular orbitals (MOs) of the chiral molecule and the MOs corresponding to the achiral configuration are related through a rigid shift from the original atomic positions to the new configuration. Orthonormality condition is enforced for both sets of wave functions, which are expressed as determinants of occupied MOs. Bellarosa *et al.*[S16] introduced the procedure to generate the achiral orbitals at the new positions, which was recently revisited and implemented within the relativistic domain.[S9,S17]

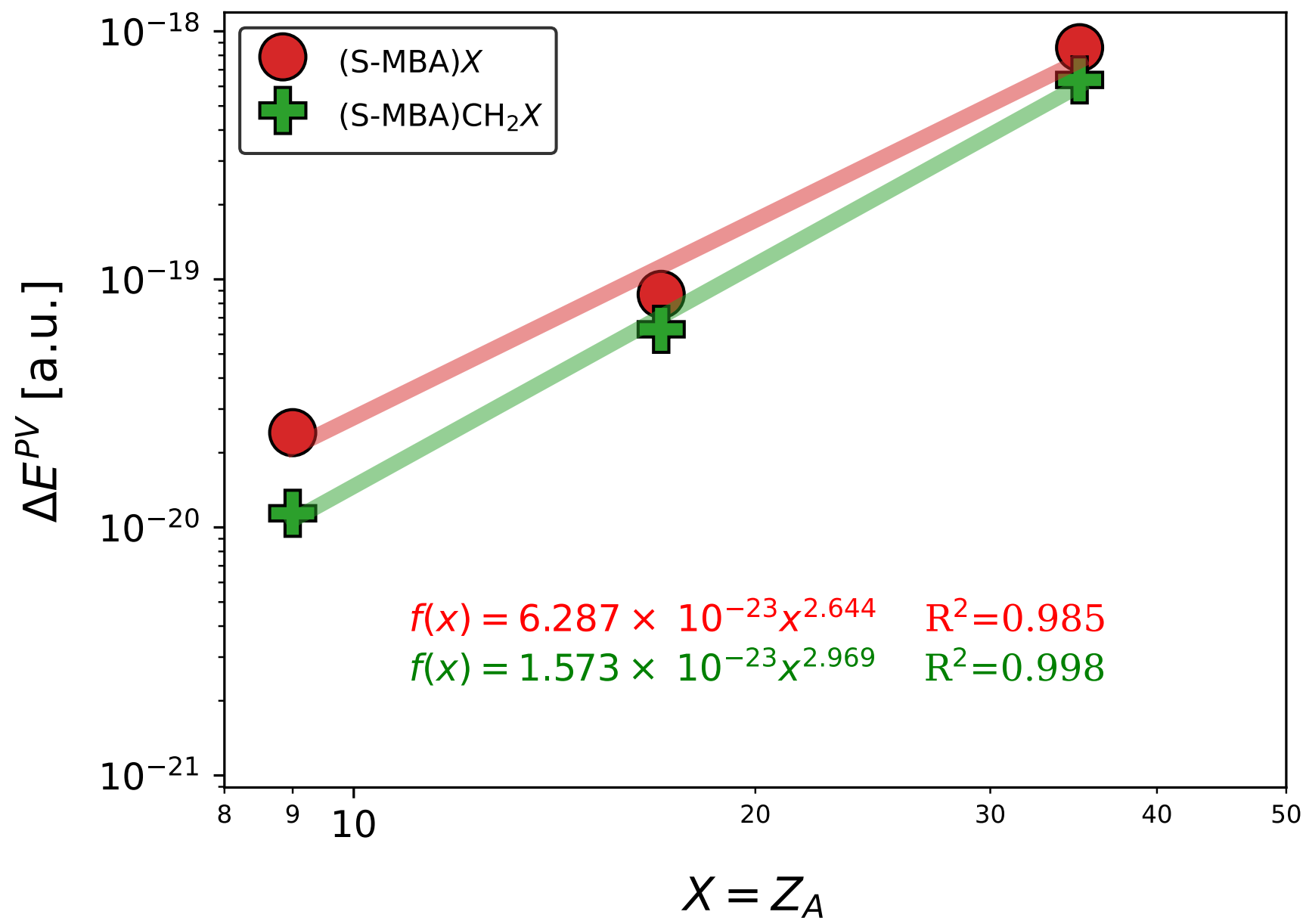

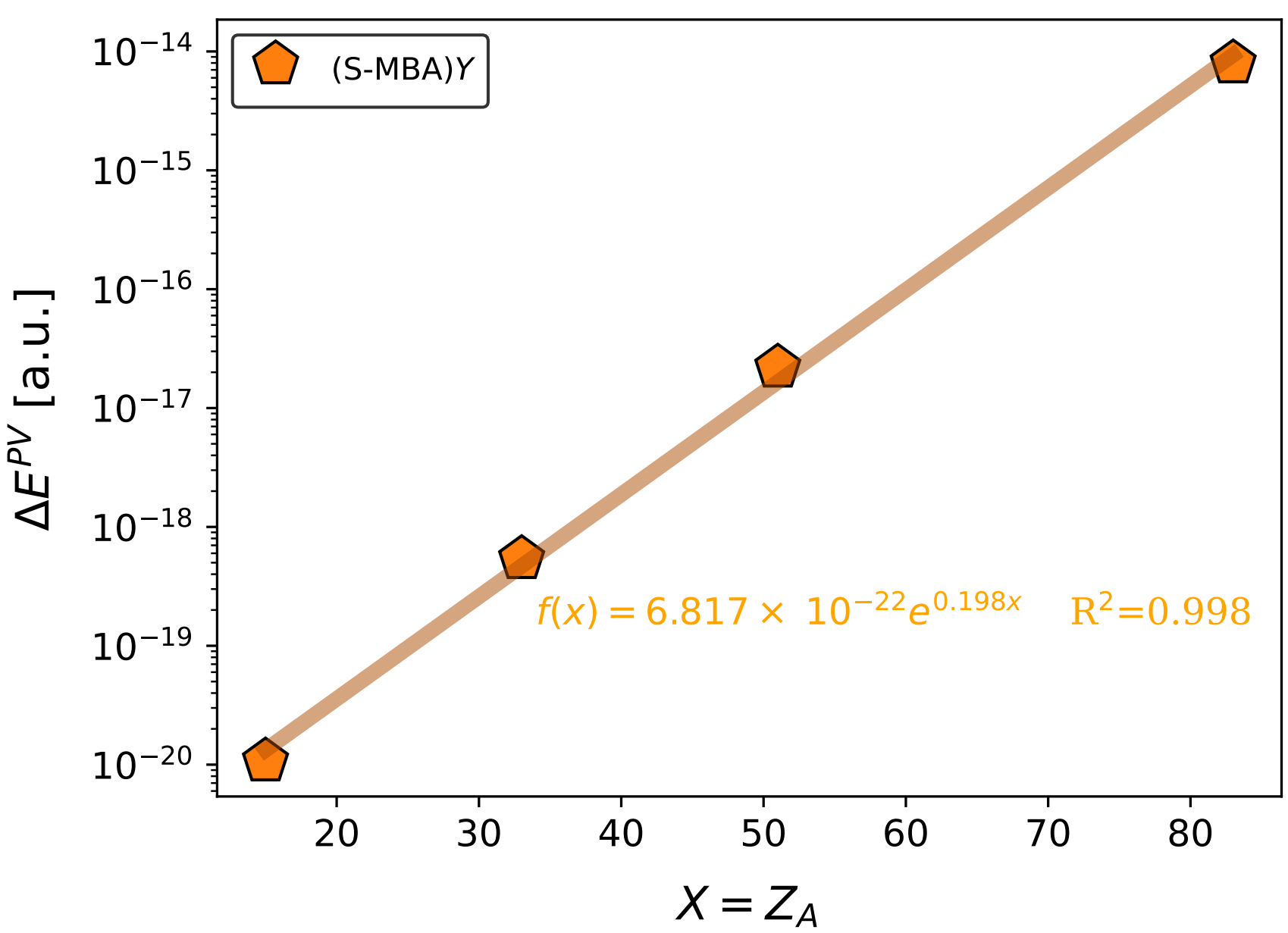

Figure S4: Correlation for ΔE^{PV} and Z_A in the two halogenated series, (S-MBA)X and (S-MBA)CH_2X (top) and for the (S-MBA)Y series (bottom). X = F, Cl, Br ; Y = P, As, Sb, Bi. The H reference points are not included in the Z_A-scaling fits. Lines are intended as a guide to the eye.

The chirality descriptor ECM is obtained as

$$ECM = \left\langle \hat{S} \right\rangle \times 100 \tag{S.3}$$

where $ECM{=}0$ corresponds to a completely achiral electronic wave function, and $ECM{=}100$ indicates no overlap between the chiral and achiral wave functions. Furthermore, when the expectation value of $\hat{Z}$ is evaluated for the chiral configuration, the transformation fully projects out the chirality information, such that a subsequent iteration cannot *extract* any additional chirality content.

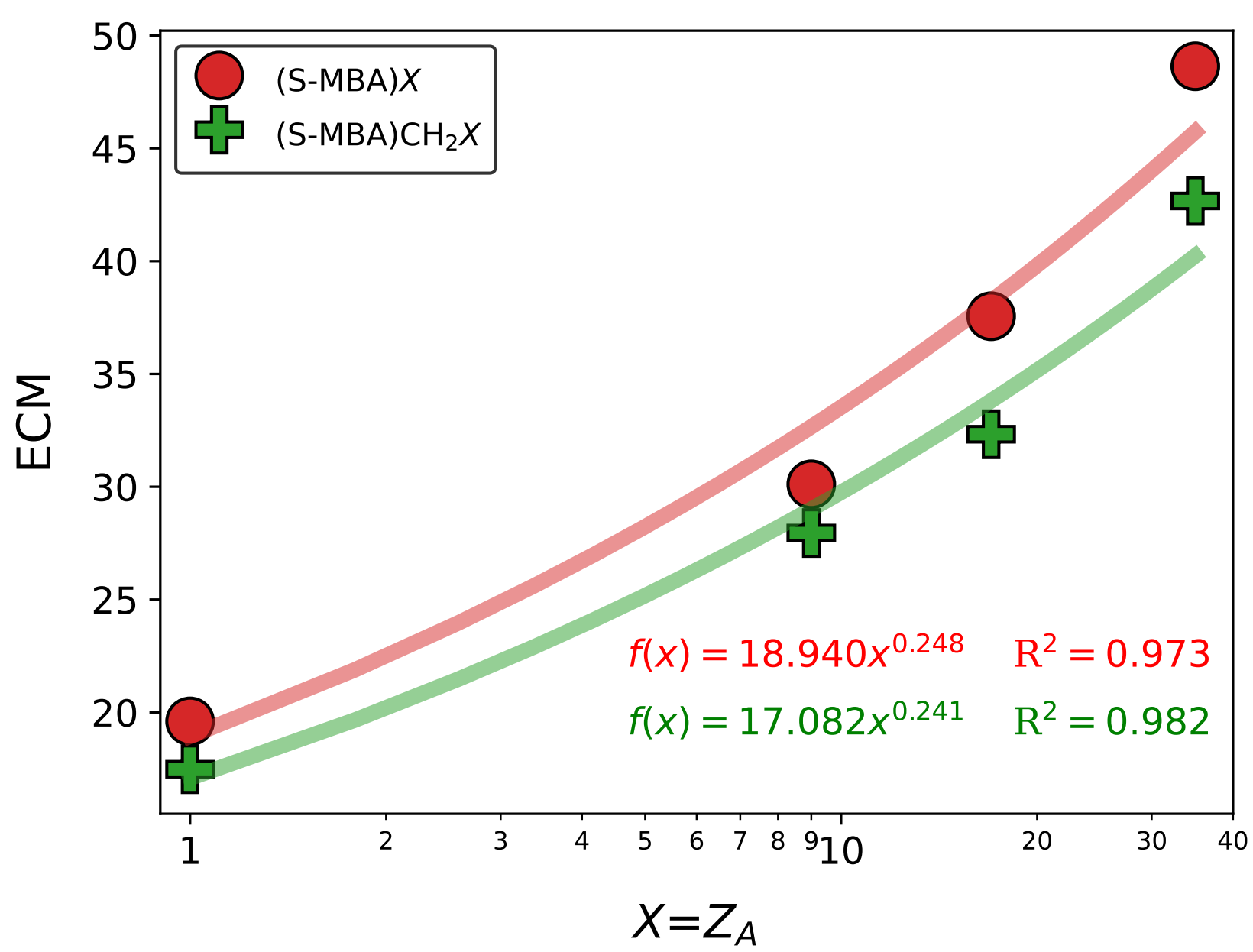

Figure S5: Correlation for ECM and the atomic number Z_A of the substituted atom in the two halogenated series, (S-MBA)X and (S-MBA)CH_2X (X = H, F, Cl, Br). Lines are intended as a guide to the eye.

Fig. S5 shows that ECM increases with the atomic number of the halogen substituent in the two halogenated series, (S-MBA)X and (S-MBA)CH_2X. No clear dependence between ECM and the atomic number of the substituted atom is observed for the (S-MBA)Y series, in contrast to the (S-MBA)X and (S-MBA)CH_2X series and our previous findings.[S9]

Effects of spin–orbit couplings in crystals

For crystals without an inversion center, the electronic energy bands are split due to the spin–orbit (SO) coupling, and the bulk SO interaction becomes odd in the electron's dipole moment $\mathbf{p}$, as observed in zinc-blende or wurtzite semiconductors. This was first described by Dresselhaus[S18] and Rashba,[S19] establishing that in 2D quantum wells with structural inversion asymmetry along the growth direction $\mathbf{z}$, the twofold spin degeneracy is lifted by the SO Hamiltonian induced by the interfacial electric field $\mathbf{E} = E_z\mathbf{z}$,[S20,S21]

$$\hat{H}_{\text{SO}}^{\text{2D}} = \alpha_R \left(\boldsymbol{\sigma} \times \boldsymbol{k}\right) \cdot \mathbf{z} \tag{S.4}$$

Here, $\mathbf{p} = \hbar\mathbf{k}$, σ_i are the Pauli matrices, $\mathbf{z}$ is a unit vector perpendicular to the surface and α_R is the Rashba parameter, with units of energy times distance. The form of Eq. (S.4) is phenomenological and derived for 2D plane waves; caution is required when applying it to realistic systems, as the lack of inversion symmetry not only creates an additional electric field E_z but also distorts the electron wavefunction close to the nuclei, where the plane-wave approximation is no longer valid[S22]

Even in the absence of external magnetic fields, electrons moving in an electric field experience an effective magnetic field in their rest frame ($\mathbf{B}_{\text{eff}} \approx \mathbf{E} \times \mathbf{p}/mc^2$) which couples to the electron's magnetic moment and induces a momentum-dependent Zeeman energy known as the SO coupling

$$\hat{H}_{\text{SO}} \approx \mu_B \mathbf{B}_{\text{eff}} \cdot \boldsymbol{\sigma} = \frac{\mu_B}{mc^2} \left(\mathbf{E} \times \mathbf{p}\right) \cdot \boldsymbol{\sigma} \tag{S.5}$$

where m and e are the electron rest mass and charge, and μ_B is the Bohr magneton.

In crystals, the effective SO magnetic field arises from the crystal's electric field. Since

SO coupling preserves time-reversal symmetry,

$$\mu_B \mathbf{B}_{\text{eff}}^{\text{SO}}(\mathbf{p}) \cdot \boldsymbol{\sigma} = -\mu_B \mathbf{B}_{\text{eff}}^{\text{SO}}(-\mathbf{p}) \cdot \boldsymbol{\sigma} \tag{S.6}$$

it follows that the SO field is odd in electron momentum, $\mathbf{B}_{\text{eff}}^{\text{SO}}(-\mathbf{p}) = -\mathbf{B}_{\text{eff}}^{\text{SO}}(\mathbf{p})$. In inversion-asymmetric crystals, SOC allows odd-in-$\mathbf{k}$ spin-dependent terms. Depending on the point-group symmetry, these terms may have Rashba-like, Dresselhaus-like, Weyl-like, or more general forms. In this work, the term Rashba-type is used only as a directional parametrization of the band-edge splitting along S–Y–S′.

The general SOC Hamiltonian for an electron moving in an intrinsic electric field can then be written as[S23,S24]

$$\hat{H}_{\text{SOC}} = \lambda(\mathbf{v} \times \mathbf{E}_{\text{chiral}}) \cdot \boldsymbol{\sigma} \tag{S.7}$$

where λ is the SOC coefficient (with units of electric charge times time), $\mathbf{v}$ is the electron velocity and $\mathbf{E}_{\text{chiral}}$ is the (local) electric field in the chiral environment. This form can be derived directly from special relativity, as discussed in the Special Relativity section. For reference, some values of the Rashba parameter in related systems can be found in Table 2 of Ref. S25.

Electric Dipole Moment

In noncentrosymmetric and, in particular, polar materials, inversion-asymmetric electric fields can influence SOC-induced band splitting. In the present chiral nonpolar crystals, we focus instead on local electrostatic fields generated by the oriented organic cations, since the total polarization is zero in $P2_12_12_1$ space group.

More recently, it was shown that in two-dimensional HOIPs a change in the orientation of the dipole moments of the organic cations corresponds to a switch in the polar axis. Consequently, by substituting iodine with bromine in the lead-halide octahedron, the polar

axis can be tuned.[S26]

In this work, we examine the relationship between the organic-cation electric dipole moment and SOC-induced band splitting. In the multipolar expansion of the electric potential for a system of charges, far from the charge density,[S27] the first non-vanishing term corresponds to the total charge Q, which is independent of the coordinate frame.[S28] However, if the coordinate origin is displaced as $\mathbf{r} \to \mathbf{r} + \mathbf{R}$, the electric dipole moment transforms as $\mathbf{p} \to \mathbf{p} + \mathbf{R}Q$. Therefore, the electric dipole moment is independent of the coordinate system only if the total charge of the system is zero.

The electronic electric dipole moment of a molecule, defined as

$$\langle \Psi | p_{e\alpha} | \Psi \rangle = -e \langle \Psi | r_\alpha | \Psi \rangle \quad , \quad \alpha = x, y, z \tag{S.8}$$

depends on the choice of the coordinate origin for charged systems. To study dipole moments of charged cations consistently,[S29] we adopt a local reference system[S26] with origin at the barycenter of the positive charges of the molecule, such that

$$\sum_{l=1}^{N} Z_l r_{l\alpha} = 0 \quad , \quad \alpha = x, y, z \tag{S.9}$$

This ensures that the nuclear contribution to the dipole moment is zero, so that $\mathbf{p} = \mathbf{p}_e$. In this way, $\mathbf{p}$ represents the electronic charge separation within the molecule and can be considered a measure of the displacement between the barycenters of positive and negative charges. Within this cation-centered convention, $\mathbf{p}_e$ in Eq. (S.8) is the intrinsic dipole descriptor reported throughout this work, and its magnitude $|\mathbf{p}|$ is given in atomic units (a.u.); for reference, 1 a.u.=2.542 D.

The SOC Hamiltonian in Eq. (S.4) is affected by the local inversion-asymmetric crystal field experienced by the Pb/I-derived band-edge states; the oriented organic cations contribute to this local electrostatic environment. The contribution from each dipole will take

the well-known expression

$$\mathbf{E}(\mathbf{r}) \approx \frac{1}{4\pi\epsilon_0}\frac{p}{r^3}\left[3(\hat{\mathbf{p}}\cdot\hat{\mathbf{r}})\hat{\mathbf{r}} - \hat{\mathbf{p}}\right] \tag{S.10}$$

where $\hat{\mathbf{p}}$ and $\hat{\mathbf{r}}$ are unit vectors centered at the dipole. This expression illustrates the leading far-field dipolar contribution; the actual local crystal field at the Pb/I band edge is obtained self-consistently in the DFT calculation.

Special relativity

The electromagnetic field tensor in contravariant form can be written as[S27]

$$F^{\mu\nu} = \begin{pmatrix} 0 & -E_x/c & -E_y/c & -E_z/c \\ E_x/c & 0 & -B_z & B_y \\ E_y/c & B_z & 0 & -B_x \\ E_z/c & -B_y & B_x & 0 \end{pmatrix} \tag{S.11}$$

where $F^{\mu\nu}$ is the electromagnetic field tensor, whose components transform covariantly under Lorentz transformations. The relativistic force acting on a particle is defined as the derivative of the four-momentum p^μ with respect to the proper time τ

$$\frac{dp^\mu}{d\tau} = qF^{\mu\nu}v_\nu \tag{S.12}$$

where v_ν is the four-velocity in covariant form. To relate the fields between two inertial frames, such as the laboratory frame and the instantaneous rest frame of the electron, the Lorentz-transformed electric and magnetic fields are

$$\mathbf{E}' = \gamma\left(\mathbf{E} + \mathbf{v}\times\mathbf{B}\right) - \frac{\gamma^2}{\gamma+1}\frac{\mathbf{v}(\mathbf{v}\cdot\mathbf{E})}{c^2} \tag{S.13}$$

$$\mathbf{B}' = \gamma\left(\mathbf{B} - \frac{\mathbf{v}\times\mathbf{E}}{c^2}\right) - \frac{\gamma^2}{\gamma+1}\frac{\mathbf{v}(\mathbf{v}\cdot\mathbf{B})}{c^2} \tag{S.14}$$

where $\gamma = \frac{1}{\sqrt{1-v^2/c^2}}$, $\mathbf{E}', \mathbf{B}'$ are the fields in the electron's frame, and $\mathbf{E}, \mathbf{B}$ correspond to the fields in the laboratory's frame. For $v \ll c$ ($\gamma \approx 1$), neglecting terms of order v^2/c^2, the transformed fields reduce to

$$\mathbf{E}' \approx \mathbf{E} + \mathbf{v} \times \mathbf{B} \tag{S.15}$$

$$\mathbf{B}' \approx \mathbf{B} - \frac{\mathbf{v} \times \mathbf{E}}{c^2} \tag{S.16}$$

These transformed fields can also be obtained directly from the Lorentz transformation of the electromagnetic field tensor,

$$F'^{\mu\nu} = \Lambda^{\mu}_{p}\Lambda^{\nu}_{\sigma}F^{p\sigma} \tag{S.17}$$

where Λ is the Lorentz transformation matrix. Rotations can easily be included in Λ, which results only in a rotation of the fields $\mathbf{E}$ and $\mathbf{B}$ without mixing them.

In particular, the effective magnetic field $\mathbf{B}_{\text{eff}}$ experienced by a moving electron in the absence of a laboratory magnetic field corresponds to $\mathbf{B}'$ with $\mathbf{B} = 0$. This effective field couples to the electron spin, giving rise to the spin–orbit interaction

$$\hat{H}_{\text{SO}} = \mu_B \boldsymbol{\sigma} \cdot \mathbf{B}_{\text{eff}} \approx -\frac{\mu_B}{c^2} \left[\boldsymbol{\sigma} \cdot (\mathbf{v} \times \mathbf{E})\right], \tag{S.18}$$

which shows that an electron moving in an intrinsic electric field experiences an effective magnetic field in its rest frame, which couples to its spin and produces momentum-dependent SOC-induced spin splitting. In the present chiral nonpolar crystals, this relativistic argument motivates the use of a Rashba-type directional energy scale, but the detailed spin texture is determined by the crystal symmetry and by the Pb/I band-edge wave functions.

References

(S1) Kroumova, E.; Aroyo, M.; Perez-Mato, J.; Ivantchev, S.; Igartua, J.; Wondratschek, H. PSEUDO: a program for a pseudosymmetry search. *J. Appl. Crystallogr.* **2001**, *34*, 783–784.

(S2) Orobengoa, D.; Capillas, C.; Aroyo, M. I.; Perez-Mato, J. M. AMPLIMODES: symmetry-mode analysis on the Bilbao Crystallographic Server. *J. Appl. Crystallogr.* **2009**, *42*, 820–833.

(S3) Flambaum, V. V.; Khriplovich, I. B. P-odd nuclear forces: a source of parity violation in atoms. *Pis'ma v Zh. Eksp. Teor. Fiz.* **1980**, *52*, 1656 [Sov. Phys. JETP 52, 835 (1980)].

(S4) Blundell, S.; Sapirstein, J.; Johnson, W. High-accuracy calculation of parity nonconservation in cesium and implications for particle physics. *Phys. Rev. D* **1992**, *45*, 1602–1623.

(S5) Hendrickson, K. R. G. Toward a measurement of atomic parity nonconservation using a single, trapped barium ion. Ph.D. thesis, University of Washington, 1999.

(S6) Tiesinga, E.; Mohr, P. J.; Newell, D. B.; Taylor, B. N. CODATA recommended values of the fundamental physical constants: 2018. *Rev. Mod. Phys.* **2021**, *93*, 025010.

(S7) Group, P. D.; Workman, R. L.; Burkert, V. D.; Crede, V.; Klempt, E.; Thoma, U.; Tiator, L.; Agashe, K.; Aielli, G.; Allanach, B. C.; et al. Review of particle physics. *Prog. Theor. Exp. Phys.* **2022**, *2022*, 083C01.

(S8) Zel'dovich, Y. B.; Saakyan, D.; Sobel'man, I. Energy difference between right-hand and left-hand molecules, due to parity nonconservation in weak interactions of electrons with nuclei. *Pis'ma v Zh. Eksp. Teor. Fiz.*. **1977**, *25*, 106 [Sov. Phys. JETP **25**, 94 (1977)].

(S9) Aucar, J. J.; Stroppa, A.; Aucar, G. A. A relationship between the molecular parity-violation energy and the electronic chirality measure. *J. Phys. Chem. Lett.* **2024**, *15*, 234–240.

(S10) Wormit, M.; Olejniczak, M.; Deppenmeier, A.-L.; Borschevsky, A.; Saue, T.; Schwerdtfeger, P. Strong enhancement of parity violation effects in chiral uranium compounds. *Phys. Chem. Chem. Phys.* **2014**, *16*, 17043–17051.

(S11) Aucar, J. J.; Maldonado, A. F. Parity violation effects on the electric field gradient. *Phys. Chem. Chem. Phys.* **2025**, *27*, 7594–7604.

(S12) Hegstrom, R.; Rein, D.; Sandars, P. Calculation of the parity nonconserving energy difference between mirror-image molecules. *J. Chem. Phys.* **1980**, *73*, 2329–2341.

(S13) Laerdahl, J. K.; Schwerdtfeger, P. Fully relativistic ab initio calculations of the energies of chiral molecules including parity-violating weak interactions. *Phys. Rev. A* **1999**, *60*, 4439–4453.

(S14) Bast, R.; Koers, A.; Gomes, A. S. P.; Iliaš, M.; Visscher, L.; Schwerdtfeger, P.; Saue, T. Analysis of parity violation in chiral molecules. *Phys. Chem. Chem. Phys.* **2011**, *13*, 864–876.

(S15) Tuvi-Arad, I.; Alon, G.; Avnir, D. CoSyM: Continuous Symmetry Measures. **2018**, http://csm.ouproj.org.il.

(S16) Bellarosa, L.; Zerbetto, F. Enantiomeric excesses and electronic chirality measure. *J. Am. Chem. Soc.* **2003**, *125*, 1975–1979.

(S17) Aucar, J. J.; Stroppa, A.; Aucar, G. A. PyECM26 (v.1.0.0). 2026; https://doi.org/10.5281/zenodo.21616439.

(S18) Dresselhaus, G. Spin-orbit coupling effects in zinc blende structures. *Phys. Rev.* **1955**, *100*, 580–586.

(S19) Rashba, E. Properties of semiconductors with an extremum loop. I. Cyclotron and combinational resonance in a magnetic field perpendicular to the plane of the loop. *Phys. Solid State* **1960**, *2*, 1109.

(S20) Vas' ko, F. Spin splitting in the spectrum of two-dimensional electrons due to the surface potential. *Pis'ma v Zh. Eksp. Teor. Fiz.* **1979**, *30*, 574 [Sov. Phys. JETP **30**, 541 (1979)].

(S21) Bychkov, Y. A.; Rashba, E. I. Oscillatory effects and the magnetic susceptibility of carriers in inversion layers. *J. Phys. C: Solid State Phys.* **1984**, *17*, 6039–6045.

(S22) Bihlmayer, G.; Koroteev, Y. M.; Echenique, P. M.; Chulkov, E. V.; Blügel, S. The Rashba-effect at metallic surfaces. *Surf. Sci.* **2006**, *600*, 3888–3891.

(S23) Manchon, A.; Koo, H. C.; Nitta, J.; Frolov, S. M.; Duine, R. A. New perspectives for Rashba spin–orbit coupling. *Nat. Mater.* **2015**, *14*, 871–882.

(S24) Wei, Q.; Zhang, Q.; Xiang, L.; Zhang, S.; Liu, J.; Yang, X.; Ke, Y.; Ning, Z. Giant spin splitting in chiral perovskites based on local electrical field engineering. *J. Phys. Chem. Lett.* **2021**, *12*, 6492–6498.

(S25) Hu, S.; Gao, H.; Qi, Y.; Tao, Y.; Li, Y.; Reimers, J. R.; Bokdam, M.; Franchini, C.; Di Sante, D.; Stroppa, A.; Ren, W. Dipole order in halide perovskites: polarization and Rashba band splittings. *J. Phys. Chem. C* **2017**, *121*, 23045–23054.

(S26) He, W.; Yang, Y.; Li, C.; Wong, W. P.; Cimpoeşu, F.; Toader, A. M.; Wu, Z.; Wu, X.; Lin, Z.; Xu, Q.-h.; Leng, K.; Stroppa, A.; Loh, K. P. Near-90 switch in the polar axis of Dion–Jacobson perovskites by halide substitution. *J. Am. Chem. Soc.* **2023**, *145*, 14044–14051.

(S27) Jackson, J. D. *Classical Electrodynamics*, 3rd ed.; Wiley: New York, 1998.

(S28) Buckingham, A. Molecular quadrupole moments. *Chem. Soc. Rev.* **1959**, *13*, 183–214.

(S29) Matar, I. K.; Matta, C. F. Origin-Dependence of Dipole Moments of Charged Proteins: Theoretical Foundations and Implications, Revisited. *J. Comput. Chem.* **2025**, *46*, e70207.
